# Reciprocal METTL3-PAX5 regulation in maintaining B-cell identity and promoting B-cell hyperreactivity in SLE

**DOI:** 10.1186/s10020-025-01295-2

**Published:** 2025-06-12

**Authors:** Yiying Yang, Ying Zhang, Shasha Xie, Ke Liu, Meidong Liu, Yisha Li, Hui Luo, Xiaoxia Zuo, Huali Zhang, Muyao Guo

**Affiliations:** 1 Department of Rheumatology, Xiangya Hospital, Department of Pathophysiology, Xiangya School of Basic Medical Sciences, Central South University, Changsha, Hunan China; 2 Sepsis Translational Medicine Key Lab of Hunan Province, Changsha, Hunan China; 3 Postdoctoral Research Station of Biology, Xiangya School of Basic Medical Sciences, Central South University, Changsha, Hunan China; 4 Provincial Clinical Research Center for Rheumatic and Immunologic Diseases, Xiangya Hospital, Central South University, Changsha, Hunan China; 5 National Clinical Research Center for Geriatric Disorders, Xiangya Hospital, Central South University, Changsha, Hunan China

**Keywords:** B cells, SLE, M6A, METTL3, PAX5

## Abstract

**Objective:**

METTL3, an m6A methyltransferase, enhances germinal center responses. This study explores its role in lupus B cells and its impact on B-cell activation.

**Methods:**

METTL3 and m6A levels in B cells from systemic lupus erythematosus (SLE) patients and lupus-prone mice were analyzed using m6A dot blot, RT-qPCR, western blotting, and flow cytometry. B-cell activation and differentiation were induced with lipopolysaccharide (LPS). The effects of METTL3 overexpression or inhibition on B-cell maturation were assessed *in vivo*. In Raji B cells, METTL3 and PAX5 knockdowns were performed to examine their regulatory relationship. EMSA and dual-luciferase assays confirmed PAX5 binding to the METTL3 promoter, while RIP and actinomycin D assays evaluated METTL3’s interaction with PAX5 mRNA. MeRIP-seq profiled m6A modifications across B-cell subsets.

**Results:**

METTL3 expression and m6A levels were significantly elevated in B cells from SLE patients, with METTL3 levels positively correlating with disease activity. Elevated m6A and METTL3 levels were observed in both naïve and activated B cells but decreased markedly during differentiation into ASCs, both *in vivo* and *in vitro*. MeRIP-seq analysis identified distinct m6A methylation patterns among B-cell subsets, particularly in key transcription factors critical for B-cell activation and differentiation. METTL3 facilitated pre-B cell development in bone marrow and maintained the balance of splenic B-cell subsets in mice. Furthermore, METTL3 preserved B-cell identity and enhanced activation. Mechanistically, METTL3 bound to *PAX5* mRNA, stabilizing it via m6A modification and promoting PAX5 expression. In turn, PAX5 directly bound to the *METTL3* promoter, driving its expression.

**Conclusion:**

The elevated expression of METTL3 in lupus B cells is linked to the maintenance of autoreactive B-cell hyperresponsiveness, contributing to the pathogenesis of SLE. The reciprocal regulation between METTL3 and PAX5 highlights a critical mechanism underlying B-cell activation and persistence in autoimmune conditions like lupus.

**Supplementary Information:**

The online version contains supplementary material available at 10.1186/s10020-025-01295-2.

## Introduction

Systemic lupus erythematosus (SLE) is a complex autoimmune disorder characterized by the production of autoantibodies due to impaired peripheral immune tolerance (Tsokos 2011). In SLE, B cells exhibit heightened activity, leading to their differentiation into autoreactive antibody-secreting cells (ASCs) that produce large amounts of autoantibodies (Rawlings et al. 2017; Lou et al. 2022; Ma et al. 2019). However, the underlying mechanisms driving B-cell hyperreactivity in SLE remain incompletely understood.

Upon antigenic stimulation, naïve B cells undergo a series of activation steps, culminating in their differentiation into ASCs. Successful B-cell activation and participation in the germinal center reaction require the maintenance of B-cell-specific transcriptional programs. In contrast, terminal differentiation into ASCs necessitates the suppression of B-cell transcriptional programs and the initiation of ASC-specific transcriptional networks (Wang et al. 2020a, 2020b; Scharer et al. 2018; Nutt et al. 2015; Shapiro-Shelef and Calame 2005). Key transcription factors, such as *Pax5 *(Cobaleda et al. 2007), *Bcl6 *(Basso and Dalla-Favera 2012), *Bach2 *(Miura et al. 2018), play pivotal roles in preserving B-cell identity and activity while inhibiting premature differentiation into ASCs.

N6-methyladenosine (m6A), the most abundant reversible post-transcriptional modification in eukaryotic mRNAs, plays a pivotal role in regulating mRNA stability, editing, and translation, thereby modulating gene expression (Sendinc and Shi 2023). The dynamic regulation of m6A is orchestrated by “writers” (e.g., METTL3, METTL14, WTAP), “erasers” (e.g., FTO, ALKBH5), and “readers” (e.g., YTHDF1, YTHDF2), which mediate the addition, removal, and interpretation of m6A marks (Huang et al. 2021; Shulman and Stern-Ginossar 2020; Barbieri and Kouzarides 2020). Recent studies have shown that m6A modifications are critical for early B-cell development, with METTL3 and METTL14 being essential for maintaining germinal center (GC) reactions (Zheng et al. 2020; Wang et al. 2023; Huang et al. 2022; Grenov et al. 2021). However, the role of m6A modifications and methyltransferases in B-cell hyperreactivity in SLE remains unclear.

In this study, we observed elevated levels of METTL3 in the B cells of lupus-prone mice and SLE patients, with these levels correlating positively with disease activity. Moreover, we found that METTL3 plays a pivotal role in preserving B-cell identity and activity following lipopolysaccharide (LPS) stimulation. Mechanistically, our data reveal that METTL3 and PAX5 reciprocally enhance each other’s expression, working collaboratively to maintain B-cell identity and promote B-cell activation. These findings provide new insights into the dysregulation of autoimmune tolerance in SLE, offering potential avenues for further investigation.

## Materials and methods

### Human samples

Ninety newly diagnosed, treatment-naïve patients diagnosed with SLE fulfilling the 2012/2019 American College of Rheumatology classification criteria (Petri et al. 2012; Aringer et al. 2019) were recruited from the Department of Rheumatology, Xiangya Hospital, Central South University. All patients were enrolled at the time of initial diagnosis and had not received any prior treatment with glucocorticoids, immunosuppressants, or biologic agents. Inclusion criteria were: (Tsokos 2011) fulfillment of the 2012 and/or 2019 ACR/EULAR classification criteria for SLE; (Rawlings et al. 2017) age between 18 and 65 years; (Lou et al. 2022) treatment-naïve status at the time of sample collection; (Ma et al. 2019) availability of complete clinical and laboratory data at diagnosis. Exclusion criteria were: (Tsokos 2011) current or recent infections (within the past 4 weeks); (Rawlings et al. 2017) coexisting autoimmune diseases or malignancies; (Lou et al. 2022) pregnancy or lactation; (Ma et al. 2019) prior use of immunosuppressive therapies, including corticosteroids or biologics. All experiments with human samples were approved by the Ethics Committee of Xiangya Hospital, Central South University (reference number:2019030465). The subjects were informed about the collection of their specimen and signed an informed consent form. The clinical information of the SLE patients were shown in Supplementary Table S1.

### Mice

C57BL/6J mice aged 6–8 weeks were purchased from the Hunan Slac Jingda Laboratory Animal Co., Ltd (Changsha, China). Mice were maintained in a specific pathogen-free (SPF) environment at the Department of Laboratory Animals, Central South University. All animal experiments were approved by the Ethical Committee for Animal Experiments of Central South University (reference number: 2020 sydw0950).

#### LPS induced B-cell activation and terminal differentiation *in vivo*

C57BL/6J mice aged 6–8 weeks were given tail vein injection of overexpressed AAV-METTL3 adeno-associated virus (Shanghai Genechem Co.,Ltd.) or intraperitoneal injection of METTL3 inhibitor STM2457 (MCE, HY-134836, 50 mg/kg). For B-cell activation and terminal differentiation *in vivo*, 50 µg LPS (Enzo Life Sciences, ALX-581-008) was administered i.v., and mice were analyzed 3 days after inoculation.

#### Pristane-induced lupus-like mouse model

Female C57BL/6J mice aged 8 weeks were intraperitoneally injected with 500 µl pristane (Sigma) per mouse. For the control group, mice were given an intraperitoneal injection of PBS following the same dosing schedule. Six months later, all mice were harvested.

### Bioinformatics analysis

The differential expression of m6A-related enzymes in transcriptome dataset GSE71698 of a subset of B cells was analyzed using *R*. B cell-specific *Mettl3* knockout dataset GSE180359 was used for differential gene expression analysis and gene set enrichment analysis (GSEA) (Subramanian et al. 2005).

### Magnetic enrichment procedures

Naive B cells were isolated from untreated mice splenocytes using immunomagnetic negative selection for CD43 (Miltenyi, 130-090−862) following the manufacturer’s protocol. Purity was confirmed by flow cytometry. ASCs were isolated from LPS treated mice splenocytes using immunomagnetic positive selection for CD138 (Miltenyi, 130-098−257) following the manufacturer’s protocol. GL7^+^ activated B cells were subsequently enriched from CD138 depleted sample. CD138 depleted cells were stained with GL7-APC (Biolegend, 144618) then enriched using a positive immunomagnetic enrichment on APC (Miltenyi, 130-090−855).

Human CD19^+^ B cells were isolated from the peripheral blood mononuclear cells (PBMCs) of healthy controls (HCs) and SLE patients by using CD19 antibody-conjugated magnetic beads (Miltenyi, 130-050−301).

### RNA m6A Immunoprecipitation and high-throughput sequencing (MeRIP-seq)

Naïve B cells, activated B cells, and ASCs were sorted by magnetic beads. Total RNA was extracted and then fragmented, and immunoprecipitated with specific anti-m6A antibody to enrich m6A-modified RNA molecules. The immunoprecipitation complex was washed to remove unspecifically bound RNA and protein, followed by elution and recovery of enriched m6A-modified RNA (Ribo Bio, riboMeRIP m6A Transcriptome Profiling Kit). Library construction was performed using the library construction Kit NEBNext^®^ utlra RNA Library Prep Kit for Illumina (NEB#E7770, USA). High-throughput sequencing was performed (Ribo Bio) using the Illumina sequencing platform PE150 mode with 6G valid data per sample. Bioinformatic analyses were then performed, including: Data quality control, rRNA Reads filtering, reference genome alignment analysis, distribution statistics of unique alignment reads, binding peak detection, binding peak annotation and statistics, motif detection and annotation of binding peaks, differential binding peak detection, differential binding peak annotation gene Gene Ontology (GO) function enrichment analysis, Kyoto encyclopedia of genes and genomes (KEGG) analysis, etc.

The Transcripts Per Kilobase of exon model per Million mapped reads (TPM) value can be obtained from the Input data. The bam file obtained by sequencing was mapped to the gtf file of the reference species using Htseq software to obtain the expression level of the transcript corresponding to the gene. edgeR was used to analyze the differential expression of genes (DEGs). Finally, the combined analysis of MeRIP-seq and RNA-seq, contains four quadrants: “m6A” and “RNA” upregulated, “m6A” and “RNA” downregulated, “m6A” upregulated and “RNA” downregulated, and “m6A” downregulated and “RNA” upregulated.

### B-cell activation and terminal differentiation *in vitro*

Naïve B cells were isolated as previously described, cultured in IMDM media (Basal media, L610 KJ) supplemented with 10% heat-inactivated fetal calf serum (Gibco, 12483020), 1x Penicillin-Streptomycin-Glutamine (NCM Biotech, C100 C5), 0.0035% β-mercaptoethanol (Sigma-Aldrich, M3148). Cells were differentiated at an initial concentration of 5 × 10^5^ cells per ml with LPS (20 µg/ml, Sigma, L2630), IL-2 (20 ng/ml, Peprotech, 212 − 12), and IL-5 (5 ng/ml, Peprotech, 212 − 15). Half doses of LPS and cytokines were given on subsequent days. The purity of B cells isolated using magnetic beads consistently exceeded 95%, as confirmed by flow cytometry (Supplementary Fig. 1).

### RNA m6A dot blot assays

Total RNA was extracted by Trizol (Accurate Biology, 21102), denatured by heating at 95°C for 3 min and transferred onto a nitrocellulose membrane (Solarbio, YA1760). The membranes were then UV cross-linked, blocked, incubated with m6A antibody (1:1000, Synaptic Systems, 202111) overnight at 4°C and subsequently incubated with HRP-conjugated goat anti-rabbit IgG (1: 5000, BOSTER, BA1055). Finally, the membranes were visualized using ECL reagents (Bio-Rad, 1705060).

### Real-time quantitative PCR (RT-qPCR)

Total RNA was reverse transcribed into cDNA with the Evo M-MLV RT Mix Kit (Accurate Biology, AG11728). qPCR analysis was performed using SYBR^®^ Green Premix (Accurate Biology, AG11701) according to the manufacturer’s instructions. The PCR primers used are listed in Supplementary Table S2.

### Western blotting (WB)

Proteins from whole cell lysates were electrophoresed on 10% SDS-polyacrylamide gels and transferred to PVDF membranes. The membranes were blocked for 1 h at room temperature. The blots were then incubated overnight at 4°C with rabbit antibodies against METTL3 (Abcam, Ab195352), METTL14 (Sigma-Aldrich, HPA038002), ALKBH5 (Sigma-Aldrich, HPA007196), and PAX5 (Abcam, ab109443). The membranes were washed three times for 10 min and then incubated for 1 h at room temperature with HRP-conjugated goat anti-rabbit IgG (BOSTER, BA1055). Finally, the membranes were visualized using ECL reagents (Bio-Rad, 1705060).

### Flow cytometry

PBMCs were prepared from the peripheral blood of HCs and SLE patients. Single-cell suspensions were prepared from the mouse spleen (SP), lymph node (LN), and bone marrow (BM). First, cells were resuspended and blocked with anti-Fc (BioLegend, 422302 human, 156604 mouse) for 10–15 min on ice. Then, Zombie Aqua fixable viability dye (BioLegend, 423106) was added to the single-cell suspensions to exclude dead cells. For surface marker detection, the cells were labeled with fluorochrome-conjugated antibodies for 30 min on ice. Intracellular staining was performed with a Fixation/Permeabilization kit (Invitrogen) following the manufacturer’s protocol. All antibodies used for flow cytometry are detailed in Supplementary Table S3.

For indirect intracellular staining of METTL3 in B cells, cells were fixed with Fixation/Permeabilization Concentrate (eBioscience, 00–5523−00) and Fixation/Perm Diluent (eBioscience, 00–5523−00) at a ratio of 1:3 for 45 min at room temperature, and then washed twice with diluted 1х Permeabilization Buffer (eBioscience, 00–5523−00). Antibody against METTL3 (Proteintech, 15073-1-AP) was added and incubated at 4℃ overnight, followed by adding the Alexa Flour 488 goat anti-rabbit IgG (Cell signaling technology, 4412) and staining for 1 h. Finally, the cells were washed twice and detected by flow cytometry (BD Biosciences).

### Raji B cell culture and transfection

Raji B cell line was purchased from the Institute for Advance Study of Central South University. The cells were cultured in RPMI 1640 media (Gibco, 72400047) supplemented with 10% heat-inactivated fetal calf serum (Gibco, 12483020) at 37°C under 5% CO2 in a humidified incubator. Raji B cells in the exponential phase were grown for 24 h and then transfected with *METTL3*-siRNA or *PAX5*-siRNA (Ribo Bio) for 48 h according to the manufacturer’s protocols.

### Electrophoretic mobility shift assay (EMSA)

Raji B cell nuclei were extracted by nuclear protein extraction kit (P0027, Beyotime, China). The binding elements of transcription factor PAX5 binding to the promoter region of the *METTL3* gene were predicted on the JASPAR website (https://jaspar.genereg.net/) and scored as shown in Table 1.

**Table 1:** Binding elements of the promoter region of the *METTL3* gene to the transcription factor PAX5

Name	Score	Relative score	Start	End	Strand	Predicted sequence
MA0014.2 METTL3	12.633	0.87515285	347	365	−1	CAGGGCAGCAAAGCAGAGT
MA0014.1 METTL3	9.700	0.81915790	1044	1063	−1	AAAGCTGTGTGGGGTGGCAG
MA0014.2 METTL3	6.567	0.80428184	1709	1727	1	AAGGGCAGAAATCTATGAT

The upstream and downstream base sequences of about 28–50 bp including the element with the highest score among the binding elements were selected for METTL3 probe design and synthesized (Table 2, Sangon Biotech, Shanghai). EMSA was performed in accordance with instructions provided in the kit (20148, Thermo Scientific™, Waltham, MA, USA), and the results were visualized using a chemiluminescence imaging system (Aplegen, USA).

**Table 2:** *METTL3* probe sequence

Binding Site	Probe	Probe sequence 5’−3’	
−347~ −365	Labeled probe	Forward	CAGCCTGGGTGACGGAGCGAGACTCCAT
		Reward	ATGGAGTCTCGCTCCGTCACCCAGGCTG
	Competitive probe	Forward	CAGCCTGGGTGACGGAGCGAGACTCCAT
		Reward	ATGGAGTCTCGCTCCGTCACCCAGGCTG
	Mutant probe	Forward	CAGCATGTGTAACAGATCGGGAATCCGT
		Reward	ACGGATTCCCGATCTGTTACACATGCTG

### Dual luciferase reporter gene assay

Luciferase reporter plasmids containing METTL3 were constructed (Bochu Biotech, Changsha, China). The plasmids were extracted (Omega, D6950-01), and the DNA was stored at −20°C for subsequent experiments. HEK293 T cells were inoculated in a 24-well plate and grown to about 70% confluence before being co-transfected (FuGENE^®^ HD, E2311) with pcDNA3.1, *PAX5* plasmids, luciferase reporter plasmids (wild type or mutant), and pRL-TK. After 48 h of transfection, the cell lysate was extracted and tested using a dual-luciferase reporter assay kit (Promega, E1910) and loaded onto a luminometer for the measurement of luminescence (SynergyHI Microplate Reader, BioTek).

### M6A-RIP-qPCR

After Raji B cells were treated with DMSO or STM2457 (MCE, HY-134836, 5 µM), the subsequent steps involved total RNA extraction, RNA fragmentation, and processing according to the m6A-RIP kit instructions (Bes5203-2(S), Bersin Bio, Guangzhou, China). An m6A-specific antibody was utilized for incubation with the fragmented RNA, allowing for the enrichment of m6A-modified RNA fragments through the specific binding of the antibody. To account for non-specific binding, an IgG antibody was employed as a negative control. RNA fragments bound to the antibodies were collected using methods such as magnetic beads, followed by elution. The eluted RNA fragments were then reverse transcribed to facilitate qPCR analysis. The primers utilized in this study are detailed in Supplementary Table S5.

### RNA immunoprecipitation (RIP)-qPCR

The RIP assay (Millipore, 17–701) was performed on Raji B cells using the METTL3 antibody according to the manufacturer’s instructions. RNA was isolated from the beads and input samples for RT-qPCR. Primers for RIP-qPCR are listed in Supplementary Table S2.

### RNA decay assay

Raji B cells were transfected with *METTL3*-siRNA (Ribo Bio) for 48 h, actinomycin D (Selleck, S8964) was added to a final concentration of 5 µM, and cells were harvested at t = 0, 1, 2, 4 h after actinomycin D treatment. Total RNAs were extracted and subjected to RT-qPCR analysis. The primers used are listed in Supplementary Table S2.

### Statistical analysis

Flow-cytometric data analysis was performed using FlowJo software (BD, USA). Clinical correlation analysis was performed with R. Unpaired Student’s t tests and two-way analysis of variance (ANOVA) were used to compare pairs of groups, and all data were presented as the Means ± SEM. *P* < 0.05 was considered statistically significant.

## Results

### METTL3 is elevated in lupus B cells and correlates with disease activity

**Fig. 1:**
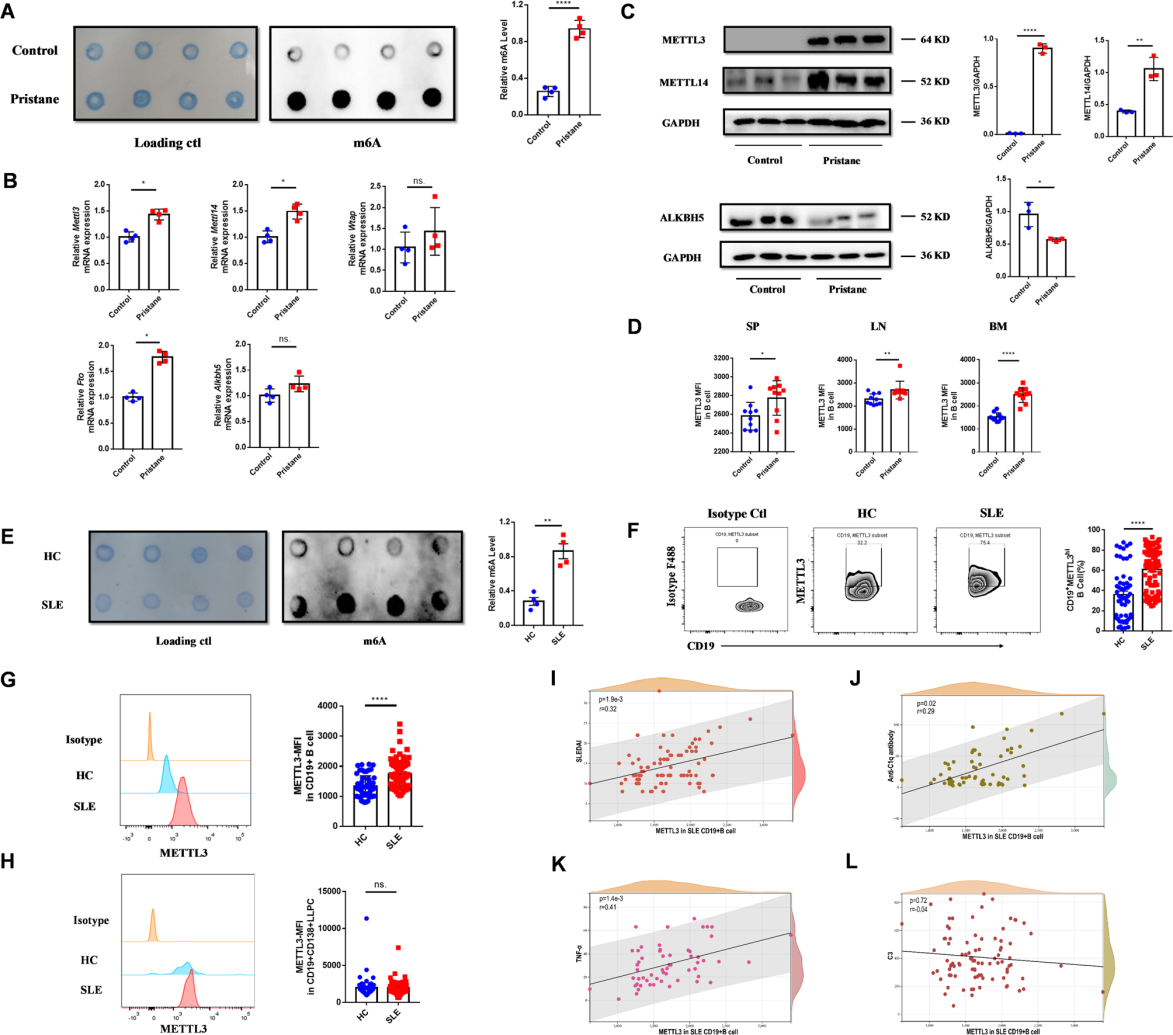
Increased METTL3 expression in lupus B cells and its correlation with disease activity. **A** M6A levels in splenic B cells of lupus mice, *n* = 4. **B** Changes in mRNA expression levels of enzymes associated with m6A modification in splenic B cells of lupus mice, *n* = 4. **C** Changes in m6A modification-related enzyme protein levels in splenic B cells of lupus mice, *n* = 3. **D** Flow cytometric analysis of elevated METTL3 expression in B cells from lupus mice, *n* = 10. **E** M6A levels in peripheral blood B cells of SLE patients, *n* = 4. **F** Flow cytometry analysis of CD19^+^METTL3^hi^ cells proportion in peripheral blood PBMCs of SLE patients and healthy controls. HC, *n* = 59; SLE, *n* = 90. **G** Flow cytometry analysis of METTL3 expression in CD19^+^B cells from peripheral blood of SLE patients and healthy controls. HC, *n* = 59; SLE, *n* = 90. **H** Flow cytometry analysis of METTL3 expression in CD19^int^D138^+^LLPCs from peripheral blood of SLE patients and healthy controls. HC, *n* = 59; SLE, *n* = 90. **I**-**L** Correlation of METTL3 expression in CD19^+^B cells of SLE patients with SLEDAI score, anti-C1q antibody levels, TNFα, and complement C3 levels SP: Spleen; LN: Lymph node; BM: Bone marrow; PBMCs: Peripheral Blood Mononuclear Cells; HC: healthy control; LLPC: long-lived plasma cells. Symbols represent individuals. Data are presented as mean ± SEM. *p < 0.05, **p < 0.01, ***p < 0.001, ****, *p* < 0.0001, ns.: no significance, by two-tailed unpaired Student’s t tests

Aberrant B-cell activation plays a critical role in the pathogenesis of SLE. Given the established importance of METTL3 in B-cell immunity (Zheng et al. 2020; Wang et al. 2023; Grenov et al. 2021), we explored its potential involvement in lupus B cells using a pristane-induced lupus mouse model (Yang et al. 2023). Our analysis revealed a significant increase in overall m6A levels in splenic B cells from lupus mice compared to controls (Fig. 1A). Western blot analysis showed elevated expression of the methyltransferases METTL3 and METTL14, and reduced expression of the demethylase ALKBH5 in lupus mice (Fig. 1B). Consistently, mRNA expression levels of *Mettl3* and *Mettl14* were upregulated, whereas *Wtap* expression remained unchanged. Notably, *Fto* mRNA levels were increased, while *Alkbh5* expression did not differ significantly from controls (Fig. 1C). Additionally, METTL3 expression was elevated in B cells from the spleen, lymph nodes, and bone marrow of lupus mice (Fig. 1D).

Similarly, peripheral blood B cells from SLE patients exhibited elevated m6A levels compared to HCs (Fig. 1E). The proportions of CD19⁺ B cells and long-lived plasma cells (LLPCs) were also increased in the peripheral blood of SLE patients relative to HCs (Supplementary Fig. 2A, B). The proportion of CD19^+^METTL3^hi^ cells in peripheral blood was significantly higher in SLE patients than in HCs (Fig. 1F), accompanied by an increase in the mean fluorescence intensity (MFI) of METTL3 in CD19^+^ B cells (Fig. 1G). However, no significant difference was observed in METTL3 expression levels in LLPCs between SLE patients and HCs (Fig. 1H).

Correlation analysis revealed that METTL3 expression in peripheral blood CD19^+^ B cells was positively associated with the SLE Disease Activity Index (SLEDAI) score (Fig. 1I), anti-C1q antibody levels (Fig. 1J), and serum tumor necrosis factor-alpha (TNFα) levels (Fig. 1K), highlighting its clinical relevance. However, no significant correlation was observed between METTL3 expression and complement C3 levels in SLE CD19^+^ B cells (Fig. 1L), despite a trend suggesting a negative association.

These findings demonstrate that METTL3 is elevated in lupus B cells and correlated with disease activity, suggesting its role in promoting aberrant B-cell activation in SLE.

### Dynamic changes in m6A and METTL3 levels during B cell activation and terminal differentiation

**Fig. 2:**
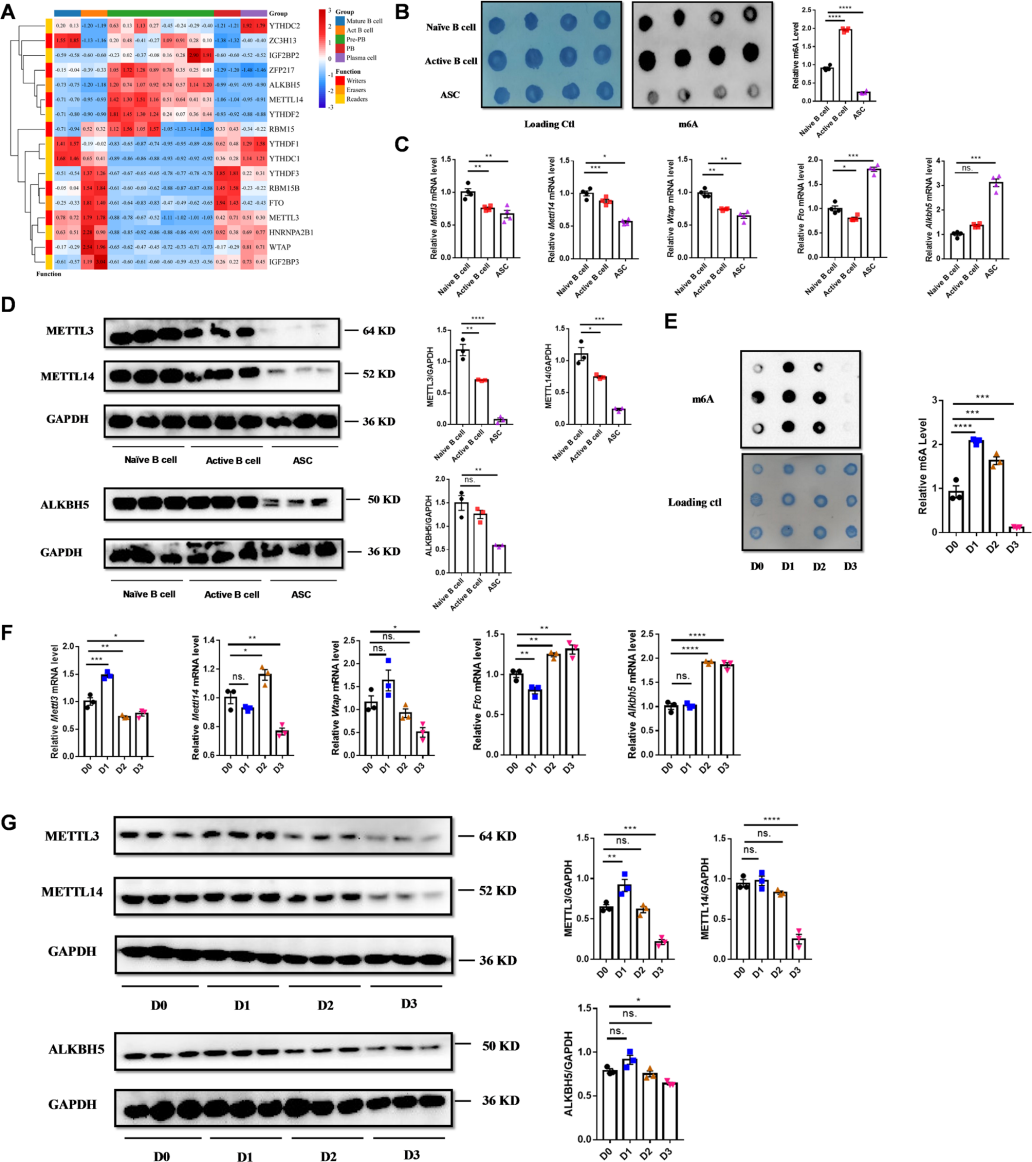
Dynamic changes in m6A and METTL3 levels during type I T-independent B-cell differentiation. **A** Heat map of gene expression related to m6A modification during B cell activation and terminal differentiation. (GSE71698). **B** Changes of m6A levels in naive B cells, activated B cells and ASCs in mice, *n* = 4. **C** Changes in mRNA expression levels of enzymes associated with m6A modification in naive B cells, activated B cells, and ASCs in mice. **D** Changes in protein levels of enzymes associated with m6A modification in naive B cells, activated B cells, and ASCs in mice. **E** Changes of m6A levels during the process of B cell activation and terminal differentiation *in vitro*. **F** Changes in mRNA expression levels of m6A modification-related enzymes during the process of B cell activation and terminal differentiation *in vitro*. **G** Changes in protein levels of m6A modification-related enzymes during the process of B cell activation and terminal differentiation *in vitro*. ASC: antibody secreting cell *n* = 3–4, symbols represent individual mice, repeated twice. Data are presented as mean ± SEM. *p < 0.05, **p < 0.01, ***p < 0.001, ****p < 0.0001, ns.: no significance, by one-way ANOVA

To elucidate the potential involvement of m6A modification in B-cell activation and terminal differentiation, we analyzed the dataset GSE71698. Bioinformatics analysis revealed significant changes in the expression profiles of multiple m6A-related genes across various B-cell subsets during *in vitro* B-cell differentiation (Fig. 2A). To further explore these findings, we stimulate T cell-independent B-cell differentiation using LPS both *in vivo* and *in vitro* (Supplementary Fig. 3).

Following *in vivo* LPS treatment, mouse naïve B cells, GL7^+^ activated B cells, and CD138^+^ ASCs were isolated for further analysis. Elevated m6A levels were observed in activated B cells compared to naïve B cells; however, these levels significantly declined in ASCs (Fig. 2B). The mRNA levels of the methyltransferases *Mettl3*, *Mettl14*, and *Wtap* were high in naïve B cells but showed a decline in activated B cells and a further reduction in ASCs. Conversely, the mRNA levels of the demethylases *Fto* and *Alkbh5* were markedly upregulated in ASCs compared to naïve B cells (Fig. 2C). Protein expression mirrored these trends for METTL3 and METTL14, with high levels in both naïve and activated B cells, but a dramatic decrease in ASCs. Interestingly, ALKBH5 protein expression did not align with its mRNA levels, as it also showed a decrease in ASCs (Fig. 2D).

To investigate m6A modifications and METTL3 expression during early B-cell activation, we performed *in vitro* stimulation of naïve B cells, which confirmed our *in vivo* findings. m6A levels significantly increased on day 1 (D1), corresponding to the early activation stage, but gradually declined, with a sharp decrease by day 3 (D3) as a substantial proportion of activated B cells differentiated into ASCs (Fig. 2E). *Mettl3* mRNA levels peaked during early activation (D1) and declined as B cells progressed to the late activation (D2) and terminal differentiation stages (D3). *Mettl14* mRNA levels remained stable during early activation (D1), increased in late activation (D2), and dropped sharply in the terminal differentiation (D3). *Wtap* mRNA levels were stable during the early (D1) and late activation (D2) stages but decreased during the terminal differentiation stage (D3). Conversely, *Fto* mRNA levels were slightly downregulated and *Alkbh5* levels remained stable at D1, but both showed upregulations at D2 and D3 (Fig. 2F). Protein levels of METTL3 and METTL14 followed similar patterns to their mRNA levels: METTL3 peaked during the early activation (D1) and declining thereafter, while METTL14 showing no significant change at D1, decreasing at D2, and reaching its lowest level at D3. ALKBH5 protein expression remained relatively stable during the early (D1) and late activation stages (D2) but slightly decreased during terminal differentiation (D3), consistent with observations in ASCs (Fig. 2G).

In summary, m6A levels and METTL3 expression increased during B-cell activation but declined during terminal differentiation, as observed both *in vivo* and *in vitro*.

### M6A modification in mRNA of key transcription factors regulating B-Cell activation and differentiation

**Fig. 3:**
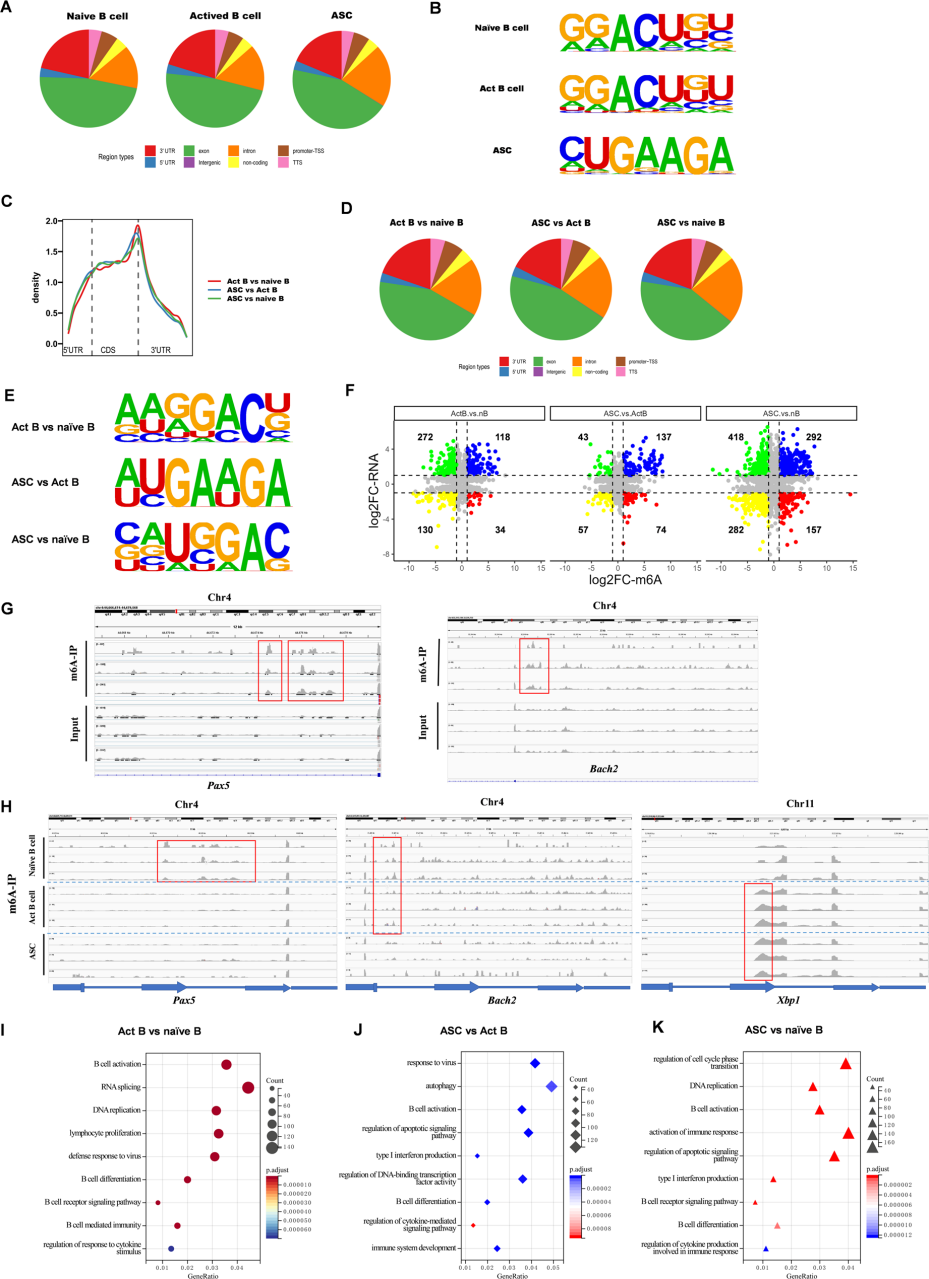
MeRIP-seq Profiling of m6A Modification Across Naïve B cells, Activated B cells, and ASCs. **A** Pie chart showing the distribution of m6A sites in seven regions of naïve B cells, activated B cells, and ASCs. **B **Top 1 motif region of naïve B cells, activated B cells, and ASCs. **C** Metagene profiles of m6A site distribution along a normalized transcript containing three rescaled non-overlapping segments: 5′ UTR, CDS, and 3′ UTR in activated B cell vs naïve B cell group, ASC vs activated B cell group, and ASC vs naïve B cell group. **D** Pie chart showing the distribution of m6A sites in seven regions of activated B cell vs naïve B cell group, ASC vs activated B cell group, and ASC vs naïve B cell group. **E** Top 1 motif region of activated B cell vs naïve B cell group, ASC vs activated B cell group, and ASC vs naïve B cell group. **F** Genes with changes in both RNA levels and m6A levels when compared across B cells (activated B cell vs naïve B cell group, ASC vs activated B cell group, and ASC vs naïve B cell group). **G **Plots of m6A peaks for *Pax5* and *Bach2* in naive B cells with m6A-IP and Input groups. **H** Plots of m6A peaks for *Pax5*, *Bach2*, and* Xbp1 *in naive B cells, activated B cell, and ASC. **I**-**K** Functional enrichment analysis of differentially expressed genes among different B cells (activated B cell vs naïve B cell group, ASC vs activated B cell group, and ASC vs naïve B cell group)

To explore how m6A methylation modulates B-cell immunity, we performed MeRIP-seq to map the global m6A landscape in naïve B cells, activated B cells, and ASCs. Bioinformatic analysis revealed that m6A peaks were predominantly located in the exon and 3’ UTR regions of mRNAs across all three cell types (Fig. 3A, B). Differential m6A peaks were significantly enriched in the 3′ UTR and near the stop codon of mRNAs when comparing activated B cells with naïve B cells, ASCs with activated B cells, and ASCs with naïve B cells (Fig. 3C-E). Stratification of m6A-modified transcripts with DEGs identified several transcripts potentially regulated by m6A methylation (Fig. 3F). For instance, compared to naïve B cells, activated B cells exhibited 272 upregulated but m6A downregulated genes (green), 118 upregulated and m6A upregulated genes (blue), 130 downregulated but m6A downregulated genes (yellow), and 34 downregulated but m6A upregulated genes (red) (Fig. 3F). A comparison of m6A-IP with input fractions revealed that several B-cell-specific transcription factors, such as *Pax5* and *Bach2*, which were highly expressed in naïve B cells, contained one or more m6A peaks (Fig. 3G). Notably, these genes exhibited dynamically changing m6A peaks across naïve B cells, activated B cells, and ASCs (Fig. 3H). GO term analysis of genes with differential m6A modifications highlighted enrichment for functions related to B cell activation, DNA replication, and type I interferon production (Fig. 3I-K).

These findings emphasize the potential regulatory role of m6A in shaping B-cell activation, particularly through the modulation of key transcription factors.

### METTL3 is essential for B-Cell maturation and homeostasis of B-Cell subsets

**Fig. 4:**
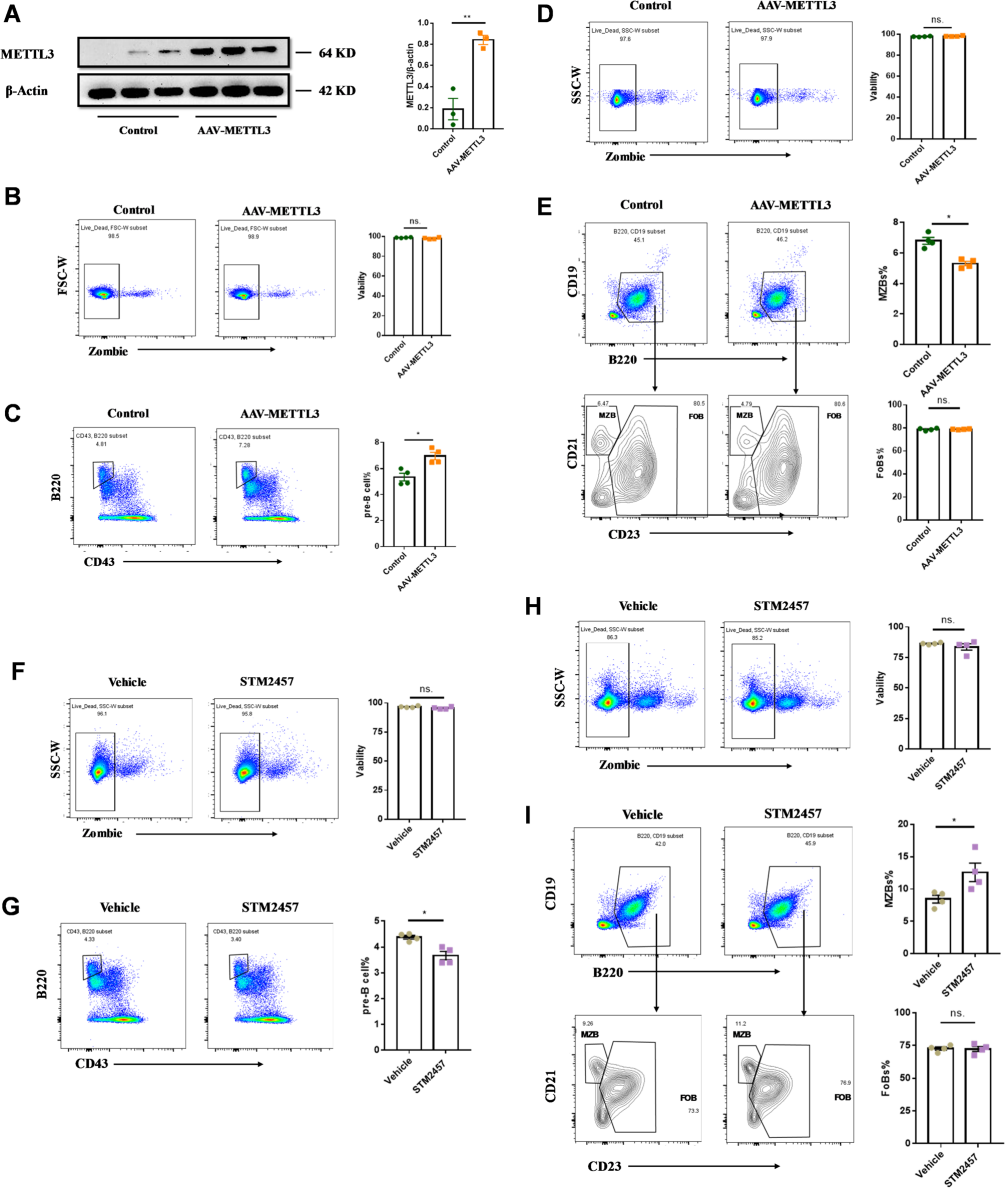
METTL3 was required for B-cell development and maintenance of B-cell subset homeostasis. **A** The effect of METTL3 overexpression in the spleen of AAV-METTL3 overexpression mice as determined by WB. **B** Flow cytometry analysis of B cell viability in mouse bone marrow following overexpression of AAV-METTL3 adeno-associated virus. **C** Flow cytometry analysis of pre-B cells proportion in the bone marrow of mice overexpressing AAV-METTL3 adeno-associated virus. **D** Flow cytometry analysis of B cell viability in the mouse spleen following overexpression of AAV-METTL3 adeno-associated virus. **E** Flow cytometry analysis of MZBs and FOBs proportions in the spleen of mice overexpressing AAV-METTL3 adeno-associated virus. **F** Flow cytometry analysis of B cell viability in mouse bone marrow following STM2457 treatment. **G** Flow cytometry analysis of pre-B cells proportion in the bone marrow of STM2457-treated mice compared to the vehicle group. **H** Flow cytometry analysis of B cell viability in the mouse spleen following STM2457 treatment. **I** Flow cytometry analysis of MZBs and FOBs proportions in the spleen of STM2457-treated mice compared to the Vehicle group MZBs: marginal zone B cells, FoBs: follicular B cells *n* = 3–4, symbols represent individual mice, repeated twice. Data are presented as mean ± SEM. *p < 0.05, **p < 0.01, ns.: no significance, by two-tailed unpaired Student’s t tests

To further explore the role of METTL3 in B-cell development and maturation, we utilized an adeno-associated virus for METTL3 overexpression and STM2457, a specific METTL3 inhibitor, to suppress its activity (Fig. 4A). Neither METTL3 overexpression nor its inhibition had a significant impact on the viability of B cells in the bone marrow or spleen of mice (Fig. 4B, D, and F, H). METTL3 overexpression increased the proportion of pre-B cells in the bone marrow of mice (Fig. 4C), and caused a slight but significant decrease in the proportion of marginal zone B cells (MZBs) in the spleen, while the percentage of follicular zone B cells (FoBs) remained unchanged (Fig. 4E). In contrast, STM2457 treatment reduced the percentage of pre-B cells in the bone marrow (Fig. 4G), while increasing the percentage of MZBs in the spleen, with the percentage of FoBs remaining stable (Fig. 4I).

Collectively, these findings highlight the role of METTL3 in promoting the early development of pro-B cells into pre-B cells in the bone marrow and maintaining the homeostasis of B-cell subsets in the spleen.

### METTL3 Inhibition suppressed B cells activation induced by LPS and CD40L

To evaluate the impact of METTL3 inhibition on splenic B cell activation, we analyzed the proportions of B220⁺CD95⁺ and B220⁺CCR6⁺ cells, along with the MFI of CD95 and CCR6, under LPS or CD40L stimulation.

**Fig. 5:**
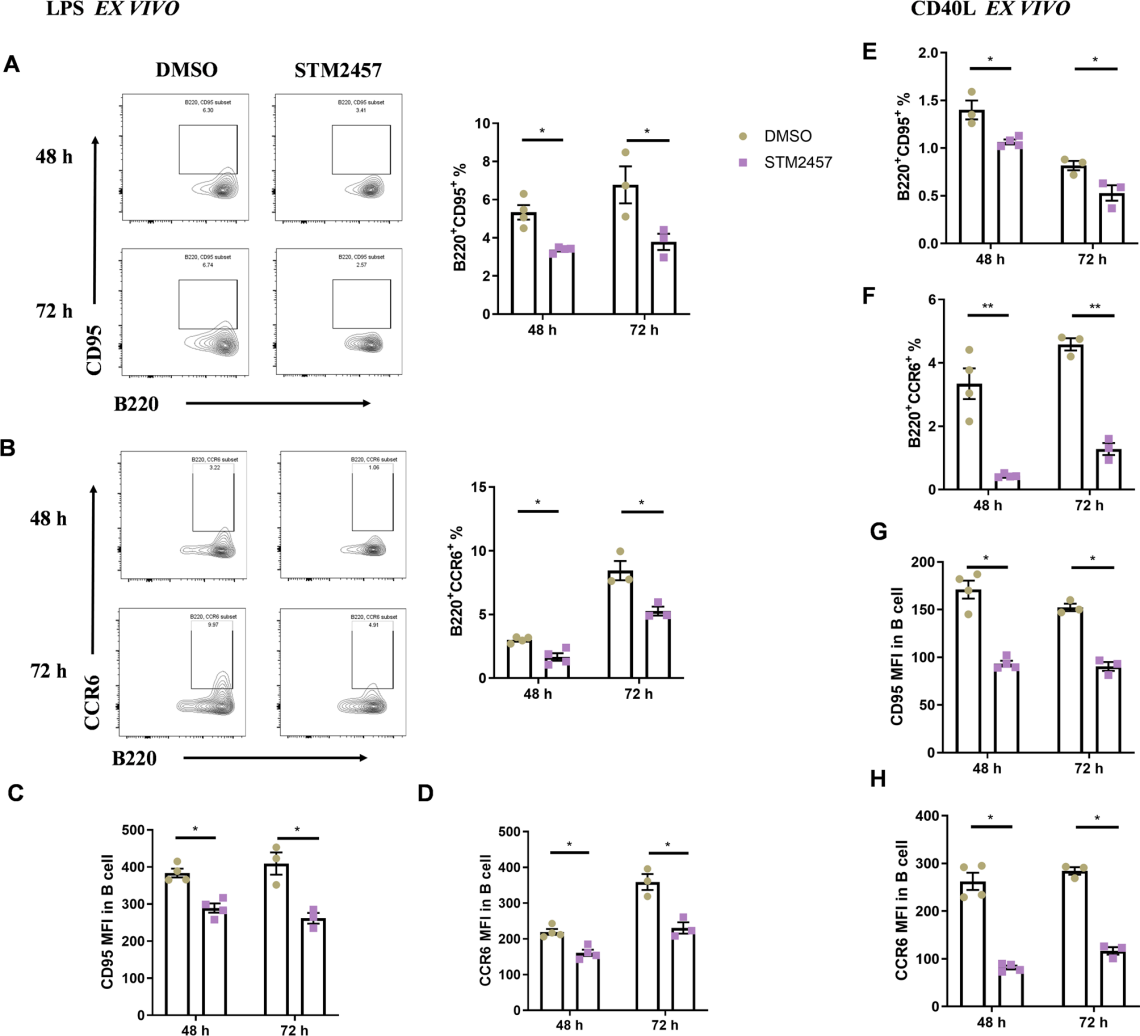
Pharmacological inhibition of METTL3 reduced early activation of splenic B cells *ex vivo*. **A**-**D** During LPS-induced activation of splenic B cells, STM2457 reduced the proportions of B220^+^CD95^+^ and B220^+^CCR6^+^ cells, and the MFI of CD95 and CCR6 in B cells were decreased after STM2457 treatment. **E**-**H** During CD40L-induced activation of splenic B cells, STM2457 reduced the proportions of B220^+^CD95^+^ and B220^+^CCR6^+^ cells, and the MFI of CD95 and CCR6 in B cells were decreased after STM2457 treatment *n* = 3–4. Data are presented as mean ± SEM. *, *p* < 0.05, **, *p* < 0.01, by two-tailed unpaired Student’s t tests

During LPS-induced activation, treatment with the METTL3 inhibitor STM2457 significantly reduced the proportions of B220⁺CD95⁺ and B220⁺CCR6⁺ cells compared to controls (Fig. 5A, B). The MFI of CD95 and CCR6 in B cells was also markedly decreased following STM2457 treatment (Fig. 5C, D).

Similarly, during CD40L-induced activation, STM2457 treatment led to a notable reduction in the proportions of B220⁺CD95⁺ and B220⁺CCR6⁺ cells (Fig. 5E, F). The MFI of CD95 and CCR6 in B cells was also significantly lower in the STM2457-treated samples than in controls (Fig. 5G, H).

These findings demonstrate that METTL3 inhibition by STM2457 effectively suppressed B cells activation induced by both LPS and CD40L stimulation.

### Reciprocal regulation between PAX5 and METTL3

To further elucidate the mechanisms by which METTL3 influences B-cell immunity, we focused on its interaction with key transcription factors. Bioinformatics analysis of transcriptomic data from GC B cells with *Mettl3* knockout (GSE180359) revealed a trend of reduced *PAX5* expression, a crucial transcription factor for maintaining B-cell identity and activation, following *Mettl3* deletion (Supplementary Fig. 4A). *Mettl3* knockout also led to alterations in pathways involved in B-cell proliferation, homeostasis, and activation (Supplementary Fig. 4B).

**Fig. 6:**
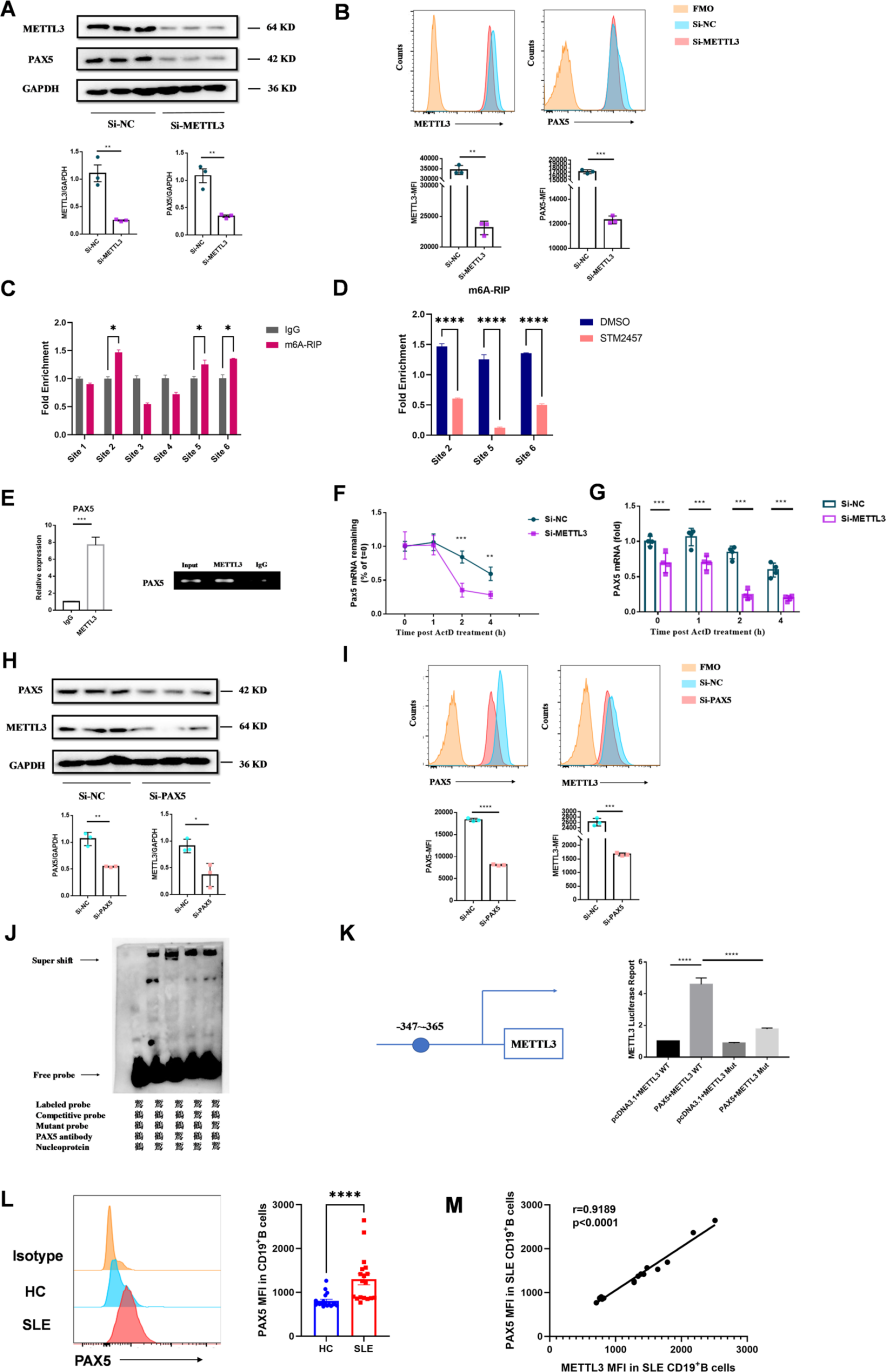
METTL3 and PAX5 interacted to maintain B-cell identity. **A** WB analysis of PAX5 protein level following *METTL3* knockdown in Raji B cells. **B** Flow cytometry analysis of PAX5 MFI after *METTL3* knockdown in Raji cells. **C** M6A-RIP-qPCR assay showing three m6A modification sites on *PAX5* mRNA. **D** M6A-RIP-qPCR assay showing that decreased m6A modification at *PAX5* mRNA upon STM2457 treatment. **E** METTL3 RIP assay showing direct binding of METTL3 to *PAX5* mRNA. **F** Actinomycin D assay to examine the effect of METTL3 on *PAX5* mRNA stability. RNA decay assay. The half-live of PAX5 mRNA was detected by quantitative RT-PCR. The remaining mRNAs were normalized to t = 0. **G** Actinomycin D assay to examine the effect of METTL3 on *PAX5* mRNA stability. Quantitative RT-PCR analysis of PAX5 mRNA abundance, relative expression was normalized to t = 0 of Si-NC cells. **H** WB analysis of METTL3 protein level following PAX5 knockdown in Raji B cells. **I** Flow cytometry analysis of METTL3 MFI after PAX5 knockdown in Raji cells. **J** EMSA showing PAX5 binding to the METTL3 gene promoter (METTL3 probe: −347 to −365). **K** Dual luciferase reporter assay detecting the effect of PAX5 on METTL3 promoter transcriptional activity. **L** Flow cytometry analysis of PAX5 expression in CD19^+^B cells from peripheral blood of SLE patients and healthy controls. HC, *n* = 18; SLE, *n* = 18. **M** Correlation of METTL3 expression with PAX5 expression in CD19^+^B cells of SLE patients Data are presented as mean ± SEM. *p < 0.05, **p < 0.01, ***, *p* < 0.001, ****, *p* < 0.0001, by two-tailed unpaired Student’s t tests

Knockdown of *METTL3* in Raji B cells significantly decreased PAX5 expression (Fig. 6A, B) and altered the mRNA levels of several B-cell-specific transcription factors and activation markers (Supplementary Fig. 5A, B). Using the SRAMP database, we identified multiple m6A modification sites on *PAX5* mRNA (Supplementary Table S4). Based on these predictions, we designed six primers pairs, selecting those with high confidence for validation (Supplementary Table S5). Subsequent m6A-RIP-qPCR results confirmed the presence of m6A modifications at the 2nd, 5 th, and 6 th sites of *PAX5* mRNA (Fig. 6C), with reduced m6A levels at these sites upon STM2457 treatment (Fig. 6D). RIP assays further validated that METTL3 direct binds to *PAX5* mRNA (Fig. 6E), and actinomycin D treatment demonstrated that *METTL3* knockdown led to a time-dependent decrease in *PAX5* mRNA levels (Fig. 6F, G). These findings indicate that METTL3 stabilizes *PAX5* mRNA through m6A modifications, thereby upregulating its expression in B cells.

Given the essential role of PAX5 as a transcription factor, we further investigated whether it transcriptionally regulates METTL3 expression in B cells. Notably, knockdown of *PAX5* in Raji B cells resulted in reduced METTL3 expression (Fig. 6H, I) and altered the mRNA levels of other enzymes involved in m6A modification (Supplementary Fig. 5C, D). Using the JASPAR database, we predicted that PAX5 binds to the *METTL3* promoter at three specific sites (Table 1). Electrophoretic mobility shift assay (EMSA) confirmed that PAX5 binds specifically to the *METTL3* promoter region between − 347 and − 365 bp (Fig. 6J). Furthermore, luciferase reporter assays demonstrated that cotransfection of *PAX5* plasmids with the wild-type METTL3 reporter significantly enhanced fluorescence, while mutations in the − 347 to −365 bp region markedly diminished this effect (Fig. 6K). These results show that PAX5 directly binds to the METTL3 promoter, upregulating its transcription.

Flow cytometry analysis revealed that the MFI of PAX5 in CD19^+^ B cells from the peripheral blood of SLE patients was significantly higher compared to that of HCs (Fig. 6L). Correlation analysis further revealed a positive association between METTL3 and PAX5 expression in CD19^+^ B cells from the peripheral blood of SLE patients (Fig. 6M).

In summary, PAX5 and METTL3 mutually enhance each other’s expression, establishing a positive feedback loop that plays a pivotal role in initiating and amplifying B-cell activation.

## Discussion

**Fig. 7:**
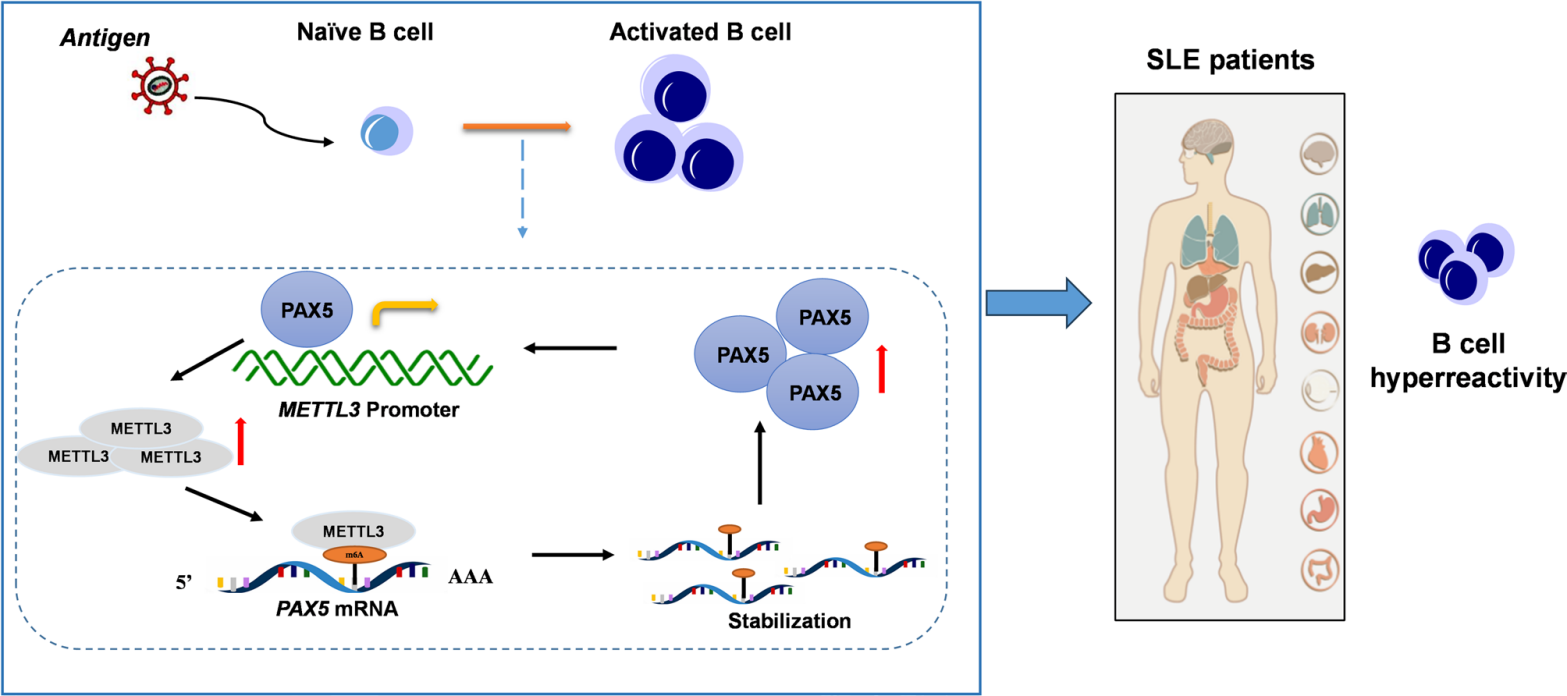
Schematic diagram showing the role of METTL3 in B-cell activation and its involvement in SLE. Elevated METTL3 and m6A levels were observed in B cells from SLE patients, correlating with disease activity. METTL3 stabilizes PAX5 mRNA through m6A modification, promoting PAX5 expression. In turn, PAX5 upregulates METTL3 in a positive feedback loop, sustaining B-cell activation. MeRIP-seq analysis revealed distinct m6A methylation patterns in B-cell subsets, which are critical for B-cell activation, contributing to SLE pathogenesis

SLE is a highly heterogeneous autoimmune disease characterized by the excessive production of autoantibodies (Yap and Chan 2019; Arbitman et al. 2022). Abnormally activated B cells play a central role in the pathogenesis of SLE. In our study, we observed significantly elevated METTL3 expression in B cells from both SLE patients and lupus mice. This increase was positively correlated with disease activity and promoted B-cell activation. Mechanistically, we found that METTL3 and PAX5 mutually enhance each other’s expression, jointly sustaining B-cell identity and contributing to the hyperreactivity characteristic of SLE (Fig. 7).

Previous studies have established that m6A modification plays a critical role in GC B-cell response (Zheng et al. 2020). METTL3 and METTL14 are essential for maintaining GC reactions, as their knockdown impairs GC formation (Huang et al. 2022; Grenov et al. 2021). Additionally, the m6A reader protein YTHDF2 promotes GC responses and facilitates B-cell activation and proliferation (Grenov et al. 2022). Our findings expand this knowledge by revealing a biphasic pattern of m6A levels and METTL3 expression during type I T cell-independent B-cell differentiation. METTL3 expression increased during early B-cell activation and declined in terminally differentiated ASCs, suggesting a role for METTL3-mediated m6A modification in maintaining B-cell identity and transcriptional programs during activation. METTL3 helps sustain the transcriptional landscape and surface marker profile characteristic of mature, functional B cells, such as expression of PAX5, thereby supporting effective clonal expansion and full activation while restraining premature differentiation into plasmablasts or immature plasma cells. Together with prior studies, our results indicate that m6A-related enzymes work in a coordinated manner to regulate B-cell transcriptional programs, thereby initiating and sustaining B-cell activation.

The process of B-cell activation and terminal differentiation is highly complex (Hagman and Lukin 2006). Despite this complexity, the transcriptional programs governing B cells and ASCs are well-defined and crucial for maintaining their distinct functions (Scharer et al. 2018; Minnich et al. 2016; Wang et al. 2020a, 2020b). Key transcription factors, including *Pax5*, *Bach2*, and *Bcl6*, orchestrate B-cell transcriptional programs, ensuring homeostasis, readiness for activation, proliferation, clonal expansion, and prevention of premature differentiation into ASCs. Elevated PAX5 levels were observed in B cells from SLE patients, contributing to enhanced survival and proliferation of autoreactive B cells—a hallmark of SLE pathogenesis. However, the mechanisms driving PAX5 upregulation and B-cell hyperactivity in SLE remain unclear. In this study, we identified that METTL3 binds to *PAX5* mRNA, facilitating its m6A modification. This modification increases the stability of *PAX5* mRNA, leading to its upregulation. These findings reveal a novel epitranscriptomic mechanism underlying the hyperresponsiveness of autoreactive B cells in lupus.

In addition to m6A modification, other epigenetic regulators contribute to the regulation of B-cell gene programs in SLE. For example, elevated EZH2, a histone methyltransferase, inhibits the expression of BACH2 and several CDK genes, thereby promoting B-cell proliferation and ASC differentiation in SLE patients (Zhang et al. 2020; Yang et al. 2022). Our previous study also demonstrated that METTL3-mediated m6A modification promotes EZH2 expression in B cells from patients with Sjögren’s disease (Yang et al. 2024). Consistently, we identified that elevated METTL3 contributes to B-cell hyperresponsiveness in lupus, suggesting a shared role of METTL3 in B-cell autoimmunity. However, the expression and function of METTL3 may vary across cell types and rheumatic diseases. In CD4^+^ T cells from SLE patients, METTL3 expression is decreased and negatively correlated with the SLEDAI score (Lu et al. 2023). Conversely, METTL3 and METTL14 are increased in salivary gland epithelial cells from patients with Sjögren’s disease, where they exhibit protective roles (Truffinet et al. 2024).

In line with previous studies showing that METTL14 is essential for B-cell development (Huang et al. 2022), our findings indicate that METTL3 overexpression increases the proportion of pre-B cells in the bone marrow, while its inhibition reduces pre-B cell populations. Elevated METTL3 mediates early B-cell development in the bone marrow and promotes peripheral B-cell immune responses in SLE pathogenesis. Additionally, we examined the impact of METTL3 on B-cell homeostasis in the spleen, focusing on B-cell viability and subtype distribution. METTL3 primarily affects the proportion of MZBs in the spleen. Since MZBs are primarily involved in T cell-independent immune responses (Palm and Kleinau 2021), this suggests that METTL3 influences B cells before their activation and differentiation.

In summary, our study identifies critical roles of METTL3 in triggering B-cell hyperactivity in SLE and in maintaining normal B-cell development and homeostasis. However, several limitations warrant further investigation. First, while we demonstrated the role of METTL3 in regulating m6A modifications and B-cell activation, the downstream effects mediated by m6A reader proteins remain unexplored. Second, studies using *Mettl3* B-cell conditional knockout mice would provide valuable insights into its therapeutic potential. Finally, the impact of METTL3 on specific B-cell subsets, such as MZBs, B1 B cells, and age-associated B cells, requires further exploration.

## Conclusion

In summary, our study identifies elevated levels of METTL3 in the B cells of lupus mice and patients, with a positive correlation to disease activity in SLE patients. METTL3 was found to promote B-cell activation. It exerted its effects by binding to *PAX5* mRNA, stabilizing it, and upregulating PAX5 expression. In turn, PAX5 was directly bound to the *METTL3* promoter, enhancing its expression and thus maintaining B-cell identity and activity. These findings offer important insights into the mechanisms underlying B-cell autoimmune tolerance defects in SLE, providing a strong basis for future exploration of targeted therapeutic strategies.

## Supplementary Information

Supplementary Material 1.

Supplementary Material 2.

Supplementary Material 3.

## Data Availability

Data is provided within the manuscript or supplementary information files. Primary data are available upon request.
