# Supplementary material for: Reciprocal METTL3-PAX5 regulation in maintaining B-cell identity and promoting B-cell hyperreactivity in SLE
Source: Mol Med. 2025 Jun 12;31:236. doi: 10.1186/s10020-025-01295-2 (PMC12160386; doi:10.1186/s10020-025-01295-2)
Supplement: Supplementary file 1 — Supplementary Material 1. [file 10020_2025_1295_MOESM1_ESM.docx]

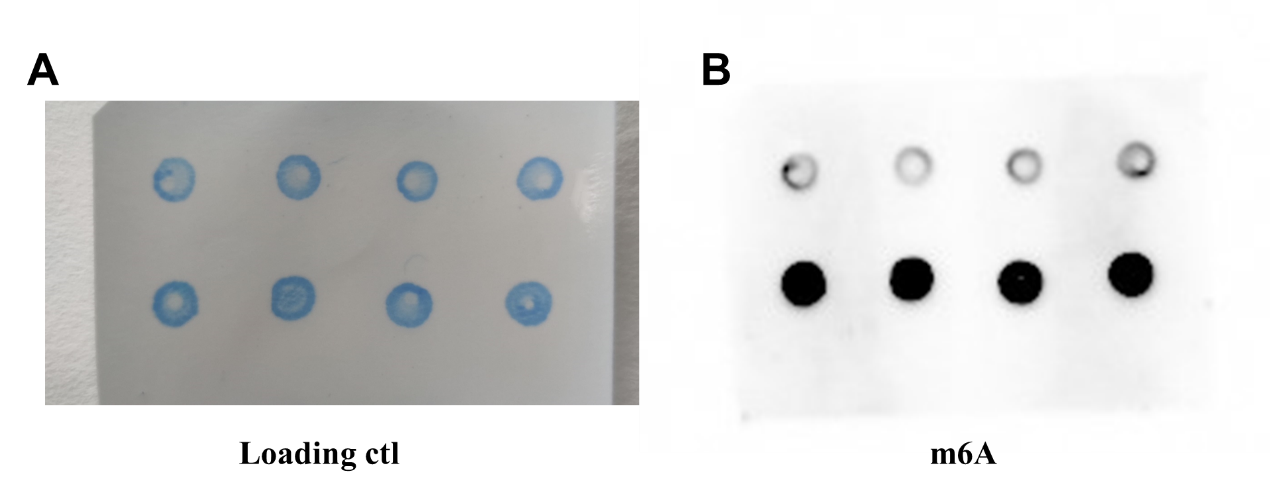


Supplementary Figure S1. The original images of the m6A dot blot assay corresponding to Figure 1A in the main text. The experiment was performed to detect the global m6A modification level in splenic B cells from pristane mice and controls. The membrane was stained with methylene blue as a loading control.
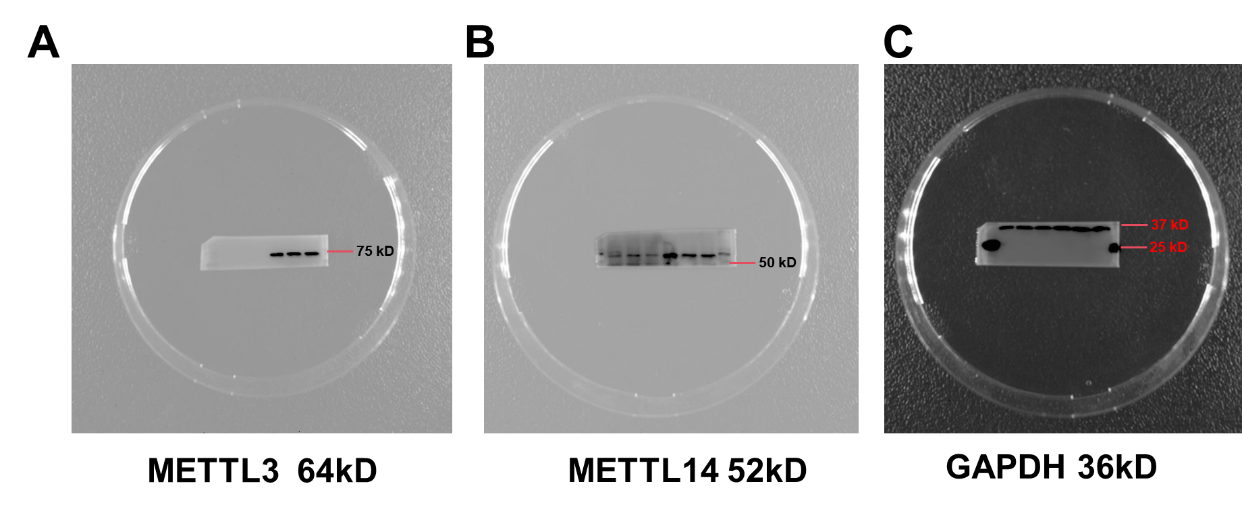


Supplementary Figure S2. The original Western blot images for METTL3 and METTL14 corresponding to Figure 1C in the main text. The full-length blots are displayed with molecular weight markers labeled. GAPDH was used as the loading control.


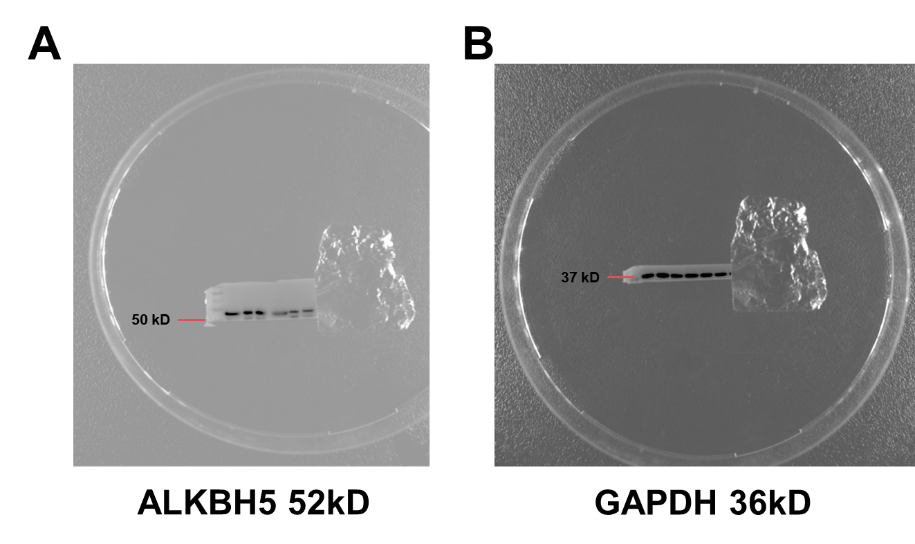


Supplementary Figure S3. The original Western blot images for ALKBH5 corresponding to Figure 1C in the main text. The full-length blots are displayed with molecular weight markers labeled. GAPDH was used as the loading control.


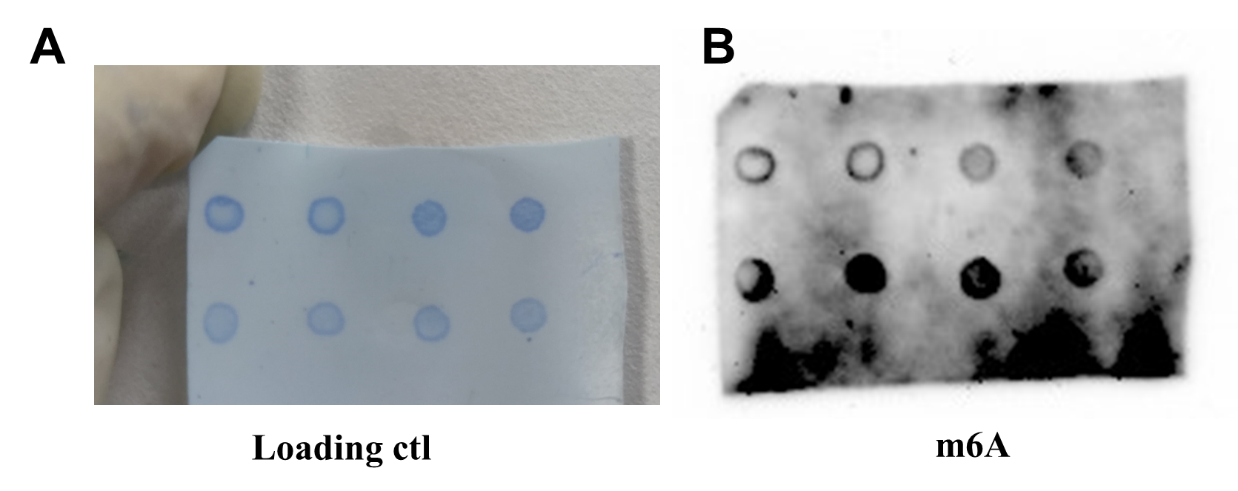


Supplementary Figure S4. The original images of the m6A dot blot assay corresponding to Figure 1E in the main text. The experiment was performed to detect the global m6A modification level in B cells from SLE patients and HCs. The membrane was stained with methylene blue as a loading control.


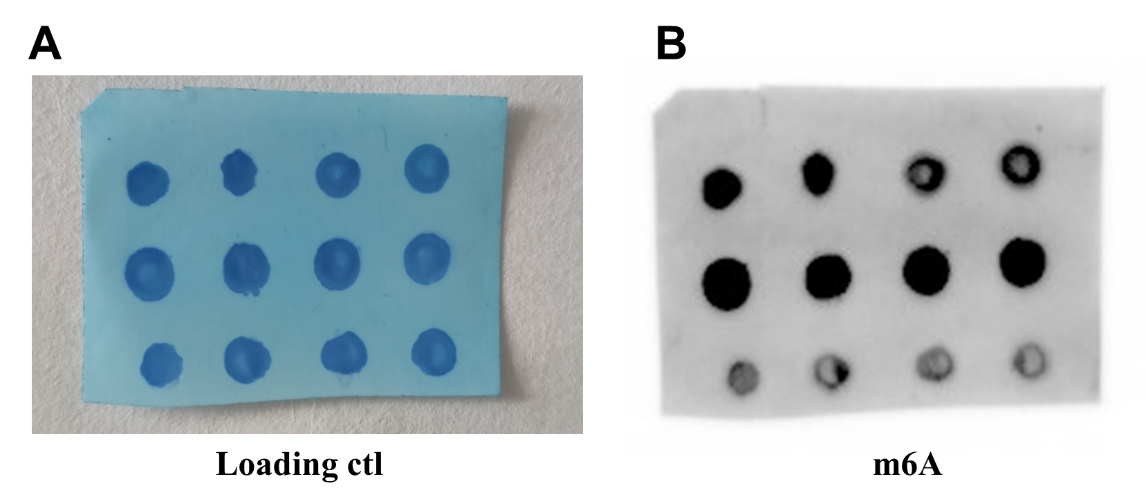


Supplementary Figure S5. The original images of the m6A dot blot assay corresponding to Figure 2B in the main text. The experiment was performed to detect the global m6A modification level in naïve B cell, active B cell, and ASC. The membrane was stained with methylene blue as a loading control.


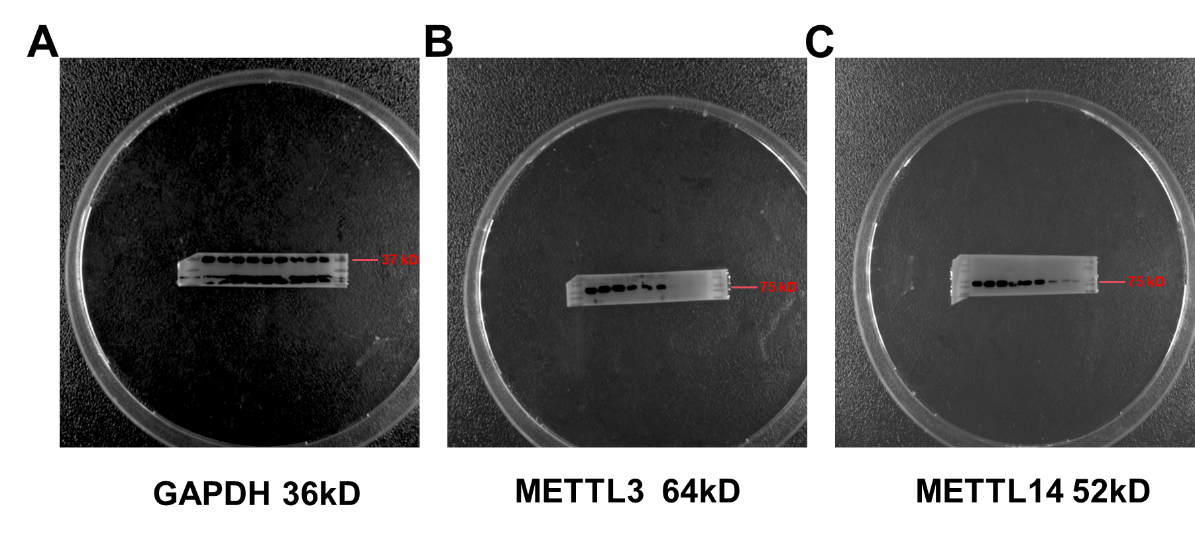


Supplementary Figure S6. The original Western blot images for METTL3 and METTL14 corresponding to Figure 2D in the main text. The full-length blots are displayed with molecular weight markers labeled. GAPDH was used as the loading control.


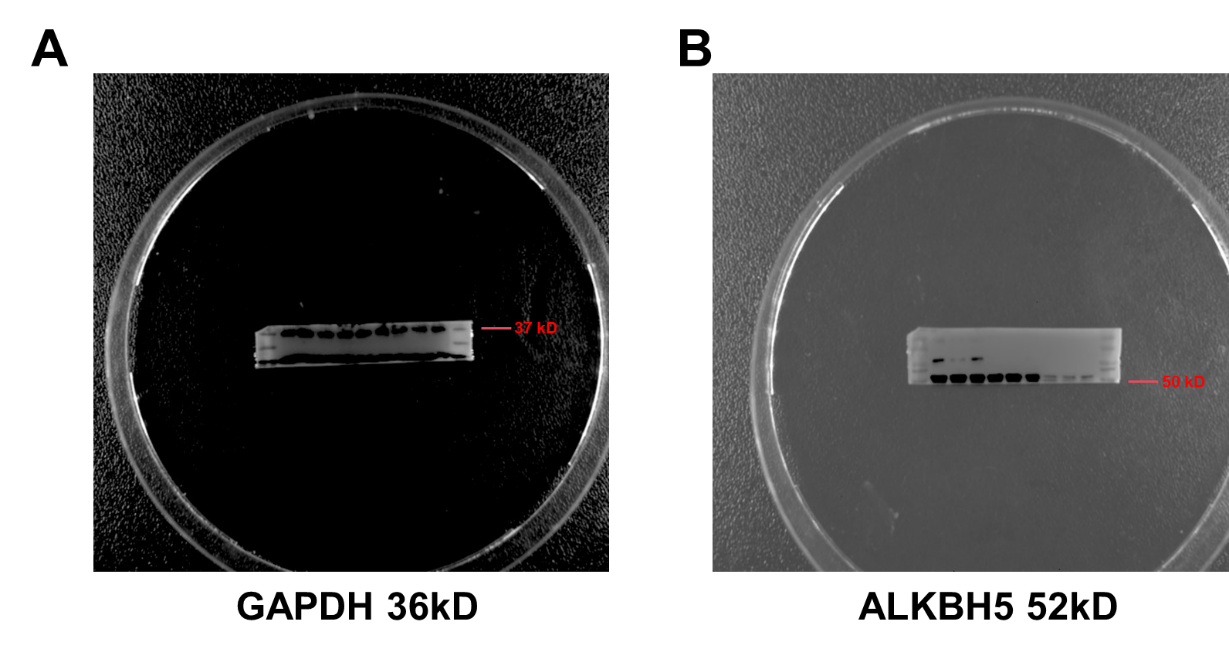


Supplementary Figure S7. The original Western blot images for ALKBH5 corresponding to Figure 2D in the main text. The full-length blots are displayed with molecular weight markers labeled. GAPDH was used as the loading control.


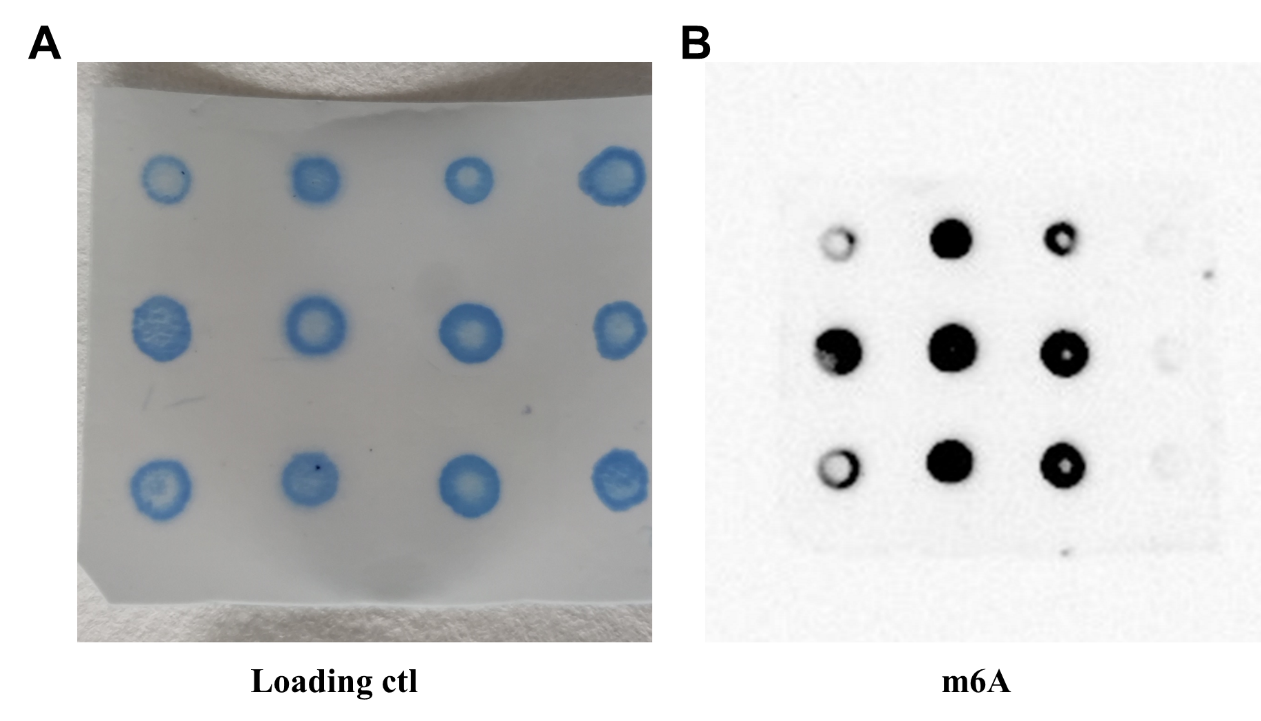


Supplementary Figure S8. The original images of the m6A dot blot assay corresponding to Figure 2E in the main text. The experiment was performed to detect the global m6A modification level in during the process of B cell activation and terminal differentiation *in vitro*. The membrane was stained with methylene blue as a loading control.


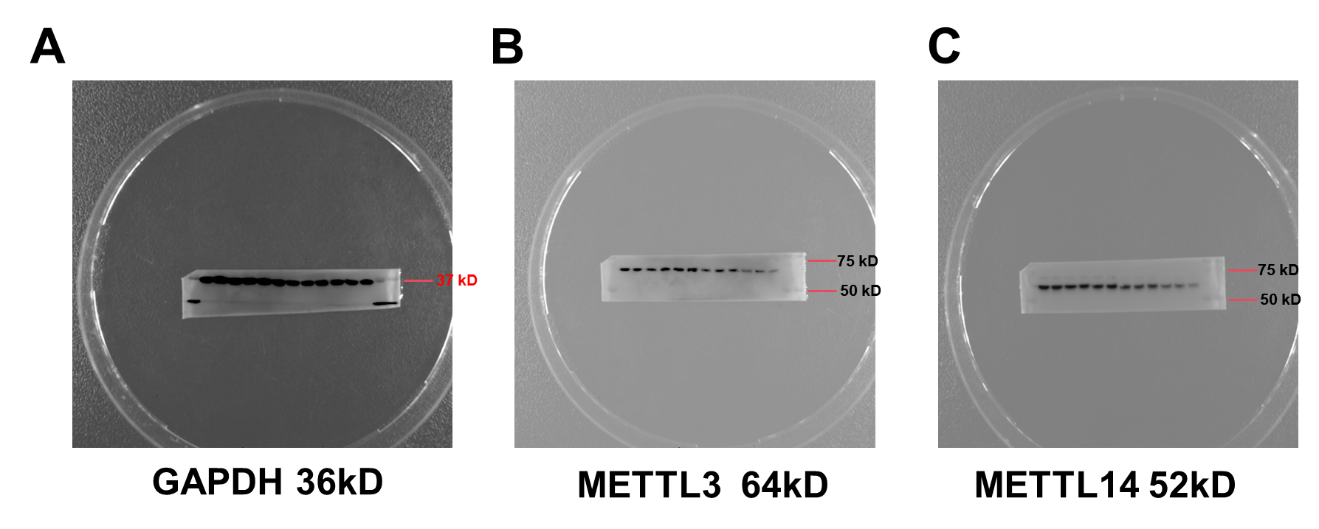


Supplementary Figure S9. The original Western blot images for METTL3 and METTL14 corresponding to Figure 2G in the main text. The full-length blots are displayed with molecular weight markers labeled. GAPDH was used as the loading control.


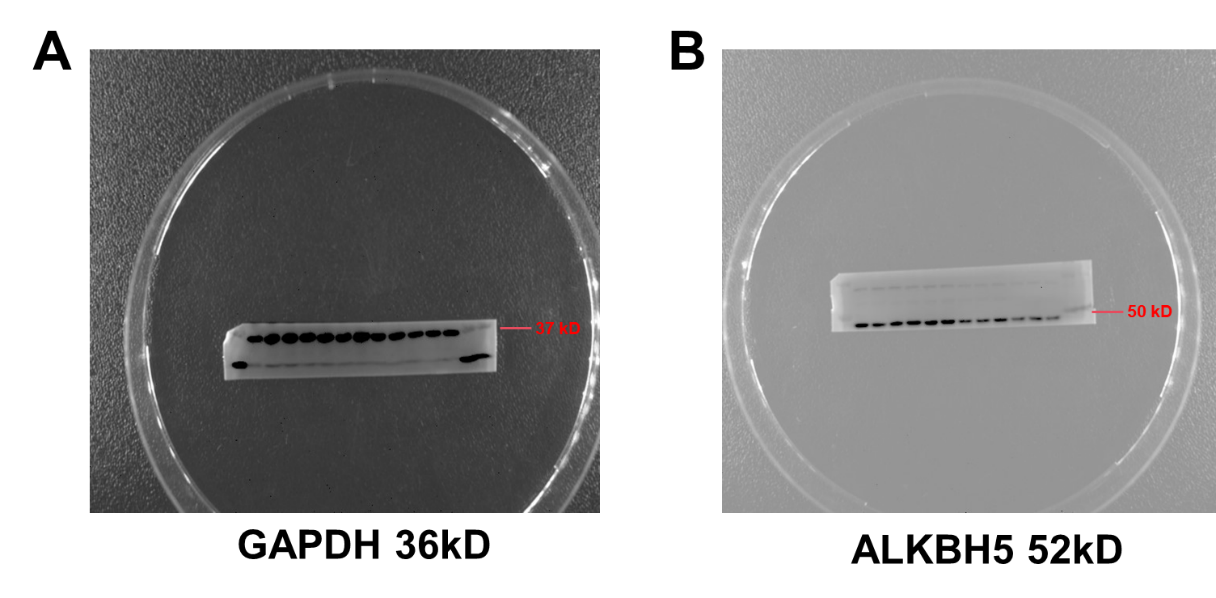


Supplementary Figure S10. The original Western blot images for ALKBH5 corresponding to Figure 2G in the main text. The full-length blots are displayed with molecular weight markers labeled. GAPDH was used as the loading control.


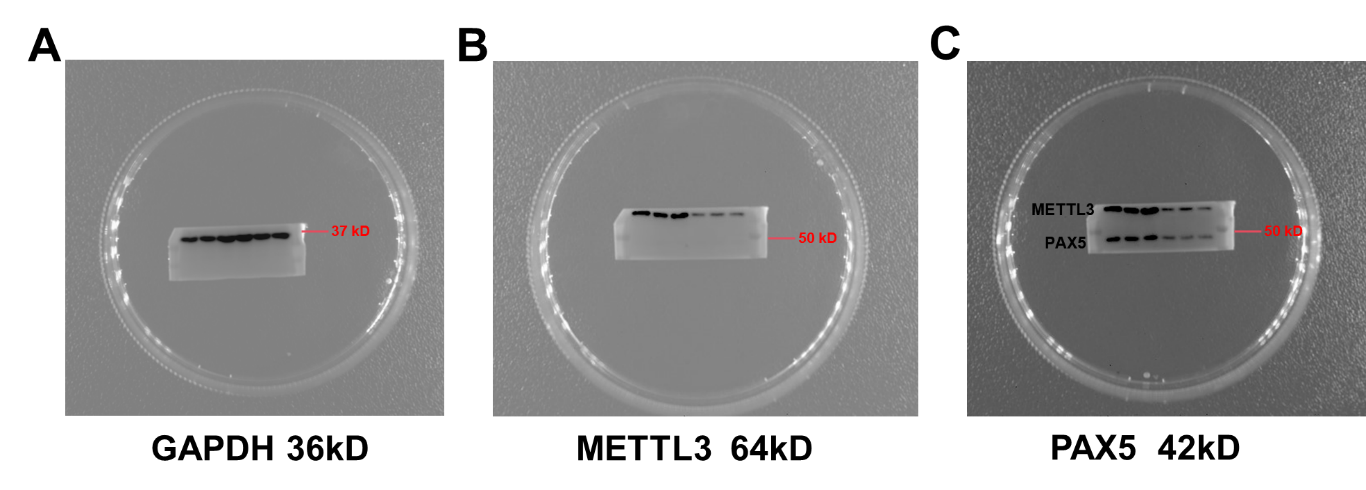


Supplementary Figure S11. The original Western blot images for METTL3 and PAX5 corresponding to Figure 6A in the main text. The full-length blots are displayed with molecular weight markers labeled. GAPDH was used as the loading control.


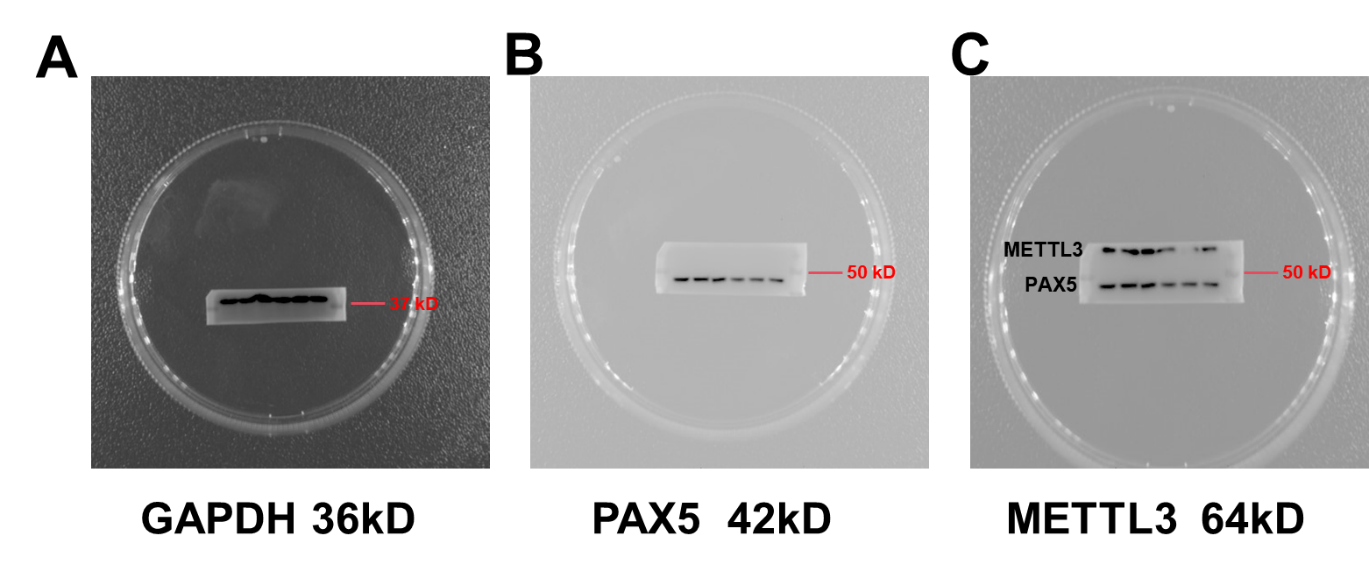


Supplementary Figure S12. The original Western blot images for METTL3 and PAX5 corresponding to Figure 6H in the main text. The full-length blots are displayed with molecular weight markers labeled. GAPDH was used as the loading control.
