# Supplementary material for: Reciprocal METTL3-PAX5 regulation in maintaining B-cell identity and promoting B-cell hyperreactivity in SLE
Source: Mol Med. 2025 Jun 12;31:236. doi: 10.1186/s10020-025-01295-2 (PMC12160386; doi:10.1186/s10020-025-01295-2)
Supplement: Supplementary file 2 — Supplementary Material 2. [file 10020_2025_1295_MOESM2_ESM.docx]

**Supplementary Table S1. The brief information of SLE patients and healthy controls.**

|  | HC (n = 59) | SLE (n = 90) | *p* value |
| --- | --- | --- | --- |
| Age | 32.9±9.1 | 33.64±13.11 | 0.7360 |
| Gender(male/female) | 4/54 | 8/82 | 0.9542 |
| SLEDAI | - | 14.29±4.73 | - |
| Disease course(months) | - | 6.44±9.72 | - |
| Fever, % | - | 29/90(32.2%) | - |
| Joint swelling and pain, % | - | 60/90(66.7%) | - |
| Facial erythema, % | - | 52/90(57.8%) | - |
| Mouth ulcers, % | - | 17/90(18.9%) | - |
| Hair loss, % | - | 19/90(21.1%) | - |
| Fatigue, % | - | 19/90(21.1%) | - |
| Photosensitivity, % | - | 5/90(5.6%) | - |
| Dry mouth, % | - | 10/90(11.1%) | - |
| Dry eyes, % | - | 5/90(6.7%) | - |
| Raynaud phenomenon, % | - | 9/90(10%) | - |
| Cough, % | - | 6/90(6.7%) | - |
| Stomach ache, % | - | 11/90(12.2%) | - |
| Diarrhoea, % | - | 8/90(8.9%) | - |
| Vomit, % | - | 10/90(11.1%) | - |
| Foamy urine, % | - | 2/90(2.2%) | - |
| Edema of both lower limbs, % | - | 13/90(14.4%) | - |
| Weight loss(>5kg), % | - | 19/90(21.1%) | - |
| WBC (×10^9^/L) | - | 3.92±2.01 | - |
| PLT (×10^9^/L) | - | 191.79±84.32 | - |
| Hb (g/L) | - | 98.74±21.65 | - |
| N (×10^9^/L) | - | 2.7±1.71 | - |
| L (×10^9^/L) | - | 0.8±0.53 | - |
| NLR | - | 4.37±3.03 | - |
| ALB (g/L) | - | 32.63±6 | - |
| GLB (g/L) | - | 35.66±8.27 | - |
| A/G | - | 0.96±0.28 | - |
| ALT (U/L) | - | 28.35±18.21 | - |
| AST(U/L) | - | 37.07±27.96 | - |
| BUN (mmol/L) | - | 5.41±2.76 | - |
| Cr (μmol/L) | - | 59.99±18.4 | - |
| ESR (mm/h) | - | 63.99±35.69 | - |
| CRP (mg/L) | - | 10.81±18.14 | - |
| IgG (g/L) | - | 21.25±7.24 | - |
| IgM (mg/L) | - | 1220.88±608.88 | - |
| IgA (mg/L) | - | 3589.12±1953.67 | - |
| C3 (mg/L) | - | 408.56±179.85 | - |
| C4 (mg/L) | - | 90.7±63.64 | - |
| Urine protein | - | 33/90 | - |
| Anti C1q antibody (U/mL) | - | 28.6±10.46 | - |
| Antinuclear antibody | - | 1:160 (8/90)  1:320 (82/90) | - |
| Anti-dsDNA antibody, % | - | 70/90(77.8%) | - |
| Anti-nRNP/Sm antibody, % | - | 51/90(56.7%) | - |
| Anti-Sm antibody, % | - | 33/90(36.7%) | - |
| Anti-SSA antibody, % | - | 64/90(71.1%) | - |
| Anti-RO52 antibody, % | - | 57/90(63.3%) | - |
| Anti-SSB antibody, % | - | 24/90(26.7%) | - |
| Anti-Scl-70 antibody, % | - | 3/90(3.3%) | - |
| Anti-centromere antibody, % | - | 0/90(0) | - |
| Anti-nucleosome antibody, % | - | 1/90(1.1%) | - |
| Anti-histone antibody, % | - | 55/90(61.1%) | - |
| Anti-ribosomal P antibody, % | - | 46/90(51.1%) | - |
| IL-1β(pg/mL) | - | 8.91±6.48 | - |
| IL-10 (pg/mL) | - | 8.8±9.18 | - |
| IL-6 (pg/mL) | - | 10.74±12.23 | - |
| TNFα(pg/mL) | - | 31.03±16.87 | - |

**Supplementary Table S2. The primers used in this study for RT-qPCR.**

| Gene | Primer sequence（5’-3’） | | | | |
| --- | --- | --- | --- | --- | --- |
| **Mouse** |  | | |  | |
| *β-Actin* | Forward | | | CTAAGGCCAACCGTGAAAG | |
|  | Reverse | | | ACCAGAGGCATACAGGGACA | |
| *Mettl3* | Forward | | | CTGGGCACTTGGATTTAAGGAA | |
|  | Reverse | | | TGAGAGGTGGTGTAGCAACTT | |
| *Mettl14* | Forward | | | CTGAGAGTGCGGATAGCATTG | |
|  | Reverse | | | GAGCAGATGTATCATAGGAAGCC | |
| *Wtap* | Forward | | | GAACCTCTTCCTAAAAAGGTCCG | |
|  | Reverse | | | TTAACTCATCCCGTGCCATAAC | |
| *Alkbh5* | Forward | | | CGCGGTCATCAACGACTACC | |
|  | Reverse | | | ATGGGCTTGAACTGGAACTTG | |
| *Fto* | Forward | | | TTCATGCTGGATGACCTCAATG | |
|  | Reverse | | | GCCAACTGACAGCGTTCTAAG | |
| **Human** |  | | |  | |
| *GAPDH* | | | Forward | CAGGAGGCATTGCTGATGAT | |
|  |  |  | Reverse | GAAGGCTGGGGCTCATTT | |
| *METTL3* | | | Forward | CATTGCCCACTGATGCTGTG | |
|  |  |  | Reverse | AGGCTTTCTACCCCATCTTGA | |
| *METTL14* | | | Forward | GAACACAGAGCTTAAATCCCCA | |
|  |  |  | Reverse | TGTCAGCTAAACCTACATCCCTG | |
| *WTAP* | | | Forward | ACTGGCCTAAGAGAGTCTGAAG | |
|  |  |  | Reverse | GTTGCTAGTCGCATTACAAGGA | |
| *ALKBH5* | | | Forward | AGTTCCAGTTCAAGCCTATTCG | |
|  |  |  | Reverse | TGAGCACAGTCACGCTTCC | |
| *FTO* | | | Forward | GCTGCTTATTTCGGGACCTG | |
|  |  |  | Reverse | AGCCTGGATTACCAATGAGGA | |
| *LRPPRC* | | | Forward | GCTCATAGGATATGGGACACACT | |
|  |  |  | Reverse | CCAGGAAATCAGTTGGTGAGAAT | |
| *IGF2BP2* | | | Forward | AGCCTGTCACCATCCATGC | |
|  |  |  | Reverse | CTTCGGCTAGTTTGGTCTCATC | |
| *YTHDF1* | | | Forward | ACCTGTCCAGCTATTACCCG | |
|  |  |  | Reverse | TGGTGAGGTATGGAATCGGAG | |
| *YTHDF2* | | | Forward | CCTTAGGTGGAGCCATGATTG | |
|  |  |  | Reverse | TCTGTGCTACCCAACTTCAGT | |
| *YTHDF3* | | | Forward | GCTATCCACCTAGTTCTCTTGGG | |
|  |  |  | Reverse | ATGCCAGGCACCTTACTCAAA | |
| *PAX5* | | | Forward | GACAATGACACCGTGCCTAGCG | |
|  |  |  | Reverse | GTGGACACTATGCTGTGACTGGAAG | |
| *BCL6* | | | Forward | GTCGTGAGGTGGTGGAGAACAAC | |
|  |  |  | Reverse | GGAATAAGAGGCTGGCGGTGTG | |
| *IRF8* | | | Forward | ACTGCTGGCTGCGTGAATGAAG | |
|  |  |  | Reverse | GTAATCGTCCACAGAAGGCTCCTTG | |
| *BACH2* | | | Forward | GCCTCCCTCTCTGTGAGTTCTCC | |
|  |  |  | Reverse | GCCTGGTTCCTGATGTTCTGTGG | |
| *MTA3* | | | Forward | TTCCCGAGACATCACCTTGTTTCAC | |
|  |  |  | Reverse | AACAGGTCCTCCGAGTGGTACTAAG | |
| *SPI.B* | | | Forward | TGGCGTCTTCTATGACCTGGACAG | |
|  |  |  | Reverse | TCCAACGGTAAGTCTTCCTCCTCTG | |
| *CD80* | | | Forward | CTCTTGGTGCTGGCTGGTCTTTC | |
|  |  |  | Reverse | AGGACAGCGTTGCCACTTCTTTC | |

**Supplementary Table S3. Antibodies used in this study.**

| Antigen | Conjugation | Isotype | Manufacturer | Clone | Cat. |
| --- | --- | --- | --- | --- | --- |
| **Mouse** |  |  |  |  |  |
| TruStain FcX™ PLUS |  | Rat IgG2b, κ | BioLegend | S17011E | 156603 |
| B220 | Alexa Fluor 488 | Rat IgG2a, κ | BioLegend | RA3-6 B2 | 103225 |
| GL7 | PE-Cy7 | Rat IgM, κ | BioLegend | GL7 | 144620 |
| GL7 | APC | Rat IgM, κ | BioLegend | GL7 | 144618 |
| CD138 | APC | Rat IgG2a, κ | BioLegend | 281-2 | 142506 |
| B220 | PE | Rat IgG2a, κ | BioLegend | RA3-6B2 | 103207 |
| GL7 | PE | Rat, IgM | Invitrogen | GL7 | 2305613 |
| B220 | PE-Cy7 | Rat IgG2a, κ | BioLegend | RA3-6B2 | 103222 |
| CD43 | PE-Cy7 | Rat IgG2b, κ | BioLegend | S11 | 143210 |
| CD23 | APC | Rat IgG2a, κ | BioLegend | B3B4 | 101620 |
| CD21 | PE | Rat IgG2a, κ | BioLegend | 7E9 | 123410 |
| CD19 | PerCP/Cy5.5 | Rat IgG2a, κ | BioLegend | 1D3/CD19 | 152406 |
| **Human** |  |  |  |  |  |
| Human TruStain FcX™ |  |  | BioLegend |  | 422302 |
| CD19 | APC-Cy7 | Mouse IgG1, κ | BioLegend | HIB19 | 302218 |
| CD138 | PerCP/Cy5.5 | Mouse IgG1, κ | BioLegend | MI15 | 356510 |
| PAX5 | PE | Mouse IgG1 | Proteintech | 2H2D6 | CL594-60349 |
| Zombie NIR™ Fixable Viability Kit | APC-Cy7 |  | BioLegend |  | 423106 |
| Anti-rabbit IgG fluorescent secondary antibody | F488 |  | Cell Signaling Technology |  | 4412 |
| Rabbit (DA1E) IgG XP® Isotype Control F(ab')2 Fragment | F488 |  | Cell Signaling Technology |  | 82131 |

**Supplementary Table S4. Prediction of m6A modification sites in Human *PAX5* mRNA**

|  | **Position** | **Sequence context** | **Decision** |
| --- | --- | --- | --- |
| 1 | 3’UTR | UAGACAAUAAUUAACACAGAGGACUUUCCCCCACACCCAGAUCAC | Very high confidence |
| 2 | 3’UTR | AGCCAGGGCAGAGAGAGACUGGACUUGGGAUCAGCAGGCCAGGCA | Very high confidence |
| 3 | 3’UTR | CUGGGUCUCUGCAAACCAAUAGACUGUCCUGCAAAUAACCGCAGC | High confidence |
| 4 | 3’UTR | GACAUUUGAUUUUGUCUUAGGGACUGACCUUUCAGCAUCAAAGAA | Very high confidence |
| 5 | 3’UTR | UUUCACCCUCUAGUGGCCUUGGACAUUGAGUAUUUGUAGAAAUGC | High confidence |
| 6 | 3’UTR | CUUUCACAUGUGCAGGGAAGAGACUCAGAUGUGGCCACAGGGCAC | High confidence |
| 7 | 3’UTR | CAGAGAGCCAGGGCAGAGAGAGACUGGACUUGGGAUCAGCAGGCC | High confidence |
| 8 | 3’UTR | CGAGGUAUUGAUUACAUUGUGGACUUUGAAUGUGAGGGCUGGAUG | High confidence |

**Supplementary Table S5. The primers of m6A binding to *PAX5* mRNA used in this study for m6A-RIP-qPCR.**

| Primer | Forward | Reverse |
| --- | --- | --- |
| *1* | CTATGAGCTTCTCCCGATGG | GCAACAAAAGCAAGCTCTCC |
| *2* | AGCAGAATGTCATCCGAGGT | AGGGAGTAGGGAGAGCCTCA |
| *3* | AGCCTGTTCCGGTGATGTAG | CCGAGAGGATGCTCAGAAGT |
| *4* | GTCACCAGGATGGCAGAGAG | GCAGGGTTTTGTCTGAGGTC |
| *5* | TTCACATGTGCAGGGAAGAG | TGGCTGAATGTCATGAGGAG |
| *6* | CCCTGGTTTTTCACCCTCTA | TGATGCTGAAAGGTCAGTCC |
