# Supplementary material for: Reciprocal METTL3-PAX5 regulation in maintaining B-cell identity and promoting B-cell hyperreactivity in SLE
Source: Mol Med. 2025 Jun 12;31:236. doi: 10.1186/s10020-025-01295-2 (PMC12160386; doi:10.1186/s10020-025-01295-2)
Supplement: Supplementary file 3 — Supplementary Material 3. [file 10020_2025_1295_MOESM3_ESM.docx]

**Supplementary Figure 1. Purity assessment of magnetically sorted B cells.**

Flow cytometry analysis confirmed a B cell purity of 98.8% following magnetic bead-based sorting.


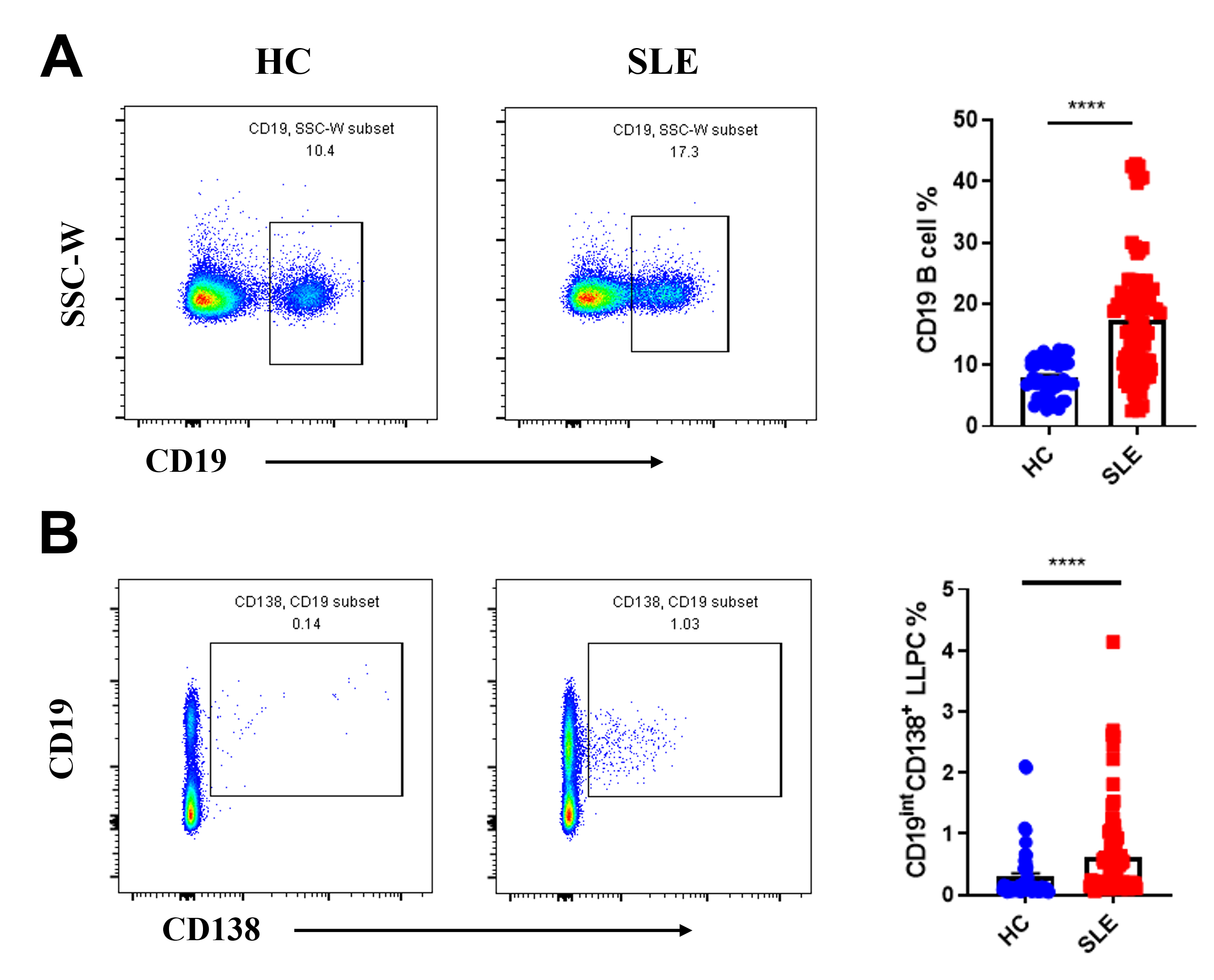


**Supplementary Figure 2. Analysis of B cells in SLE patient PBMCs.**

1. Representative flow cytometry plot and scatter plot showing the relative frequency of CD19^+^B cells% in PBMC from SLE patients.
2. Representative flow cytometry plot and scatter plot showing the relative frequency of CD19^int^ CD138^+^ LLPC% in PBMC from SLE patients.

HC, n = 59; SLE, n = 90. HC: healthy control; LLPC: long-lived plasma cells. Symbols represent individuals. Data are presented as mean ± SEM. ****, *p* < 0.0001, ns.: no significance, by two-tailed unpaired Student’s t tests.


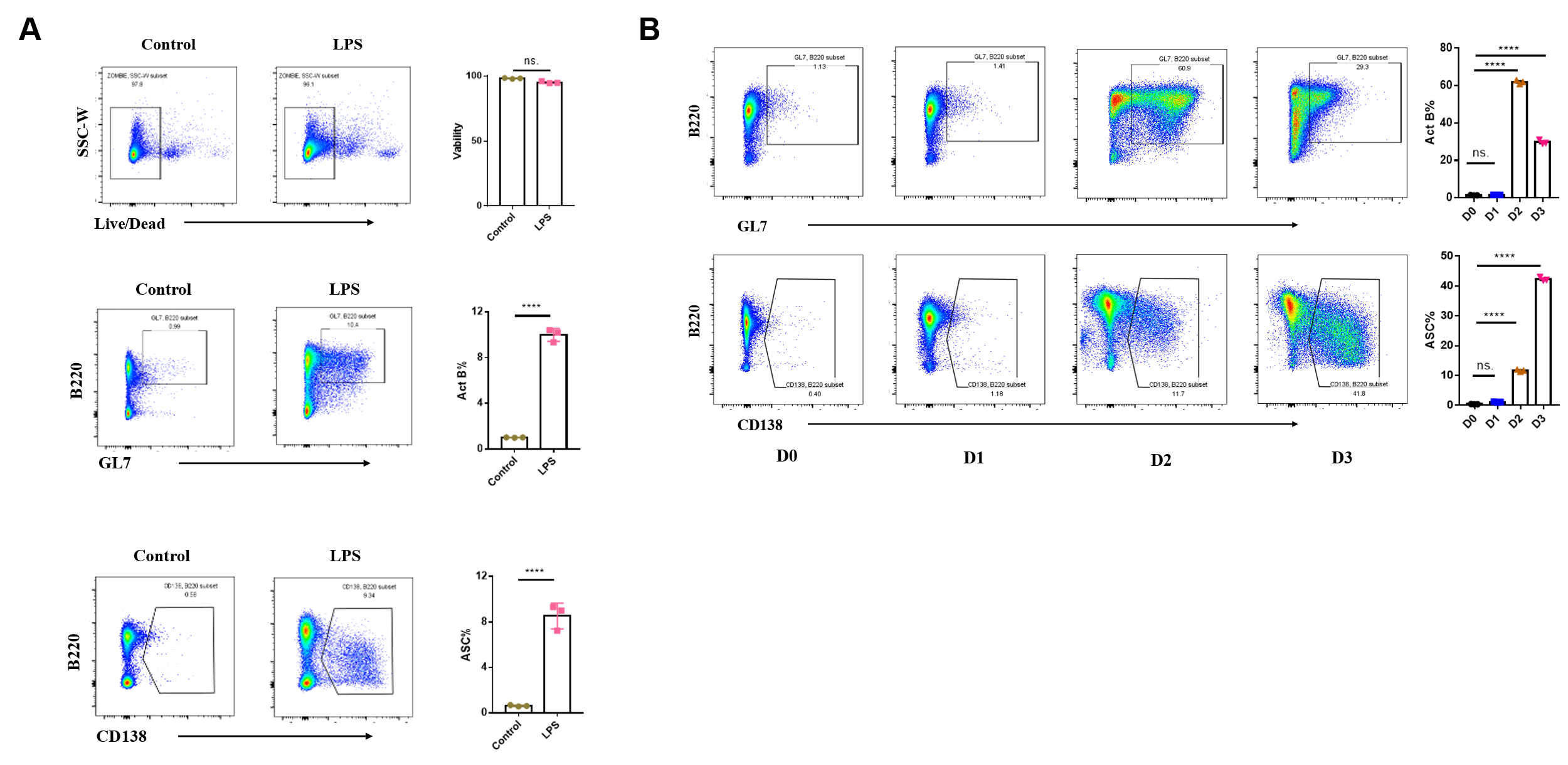


**Supplementary Figure 3. LPS induces activation and terminal differentiation of B cells *in vivo* and *in vitro*.**

A. LPS induces activation and terminal differentiation of B cells *in vivo*. Splenocyte viability of mice in LPS group and control group. The proportion of activated B cells (B220^+^GL7^+^) and ASCs (B220^int^CD138^+^) in the spleen of LPS group and control group.

B. Process of activation and differentiation of mouse B cells *in vitro*. Proportion of activated B cells and ASCs on different days of culture *in vitro*.

n = 3. Data are presented as mean ± SEM. ****, *p* < 0.0001, ns.: no significance, by two-tailed unpaired Student’s t tests.


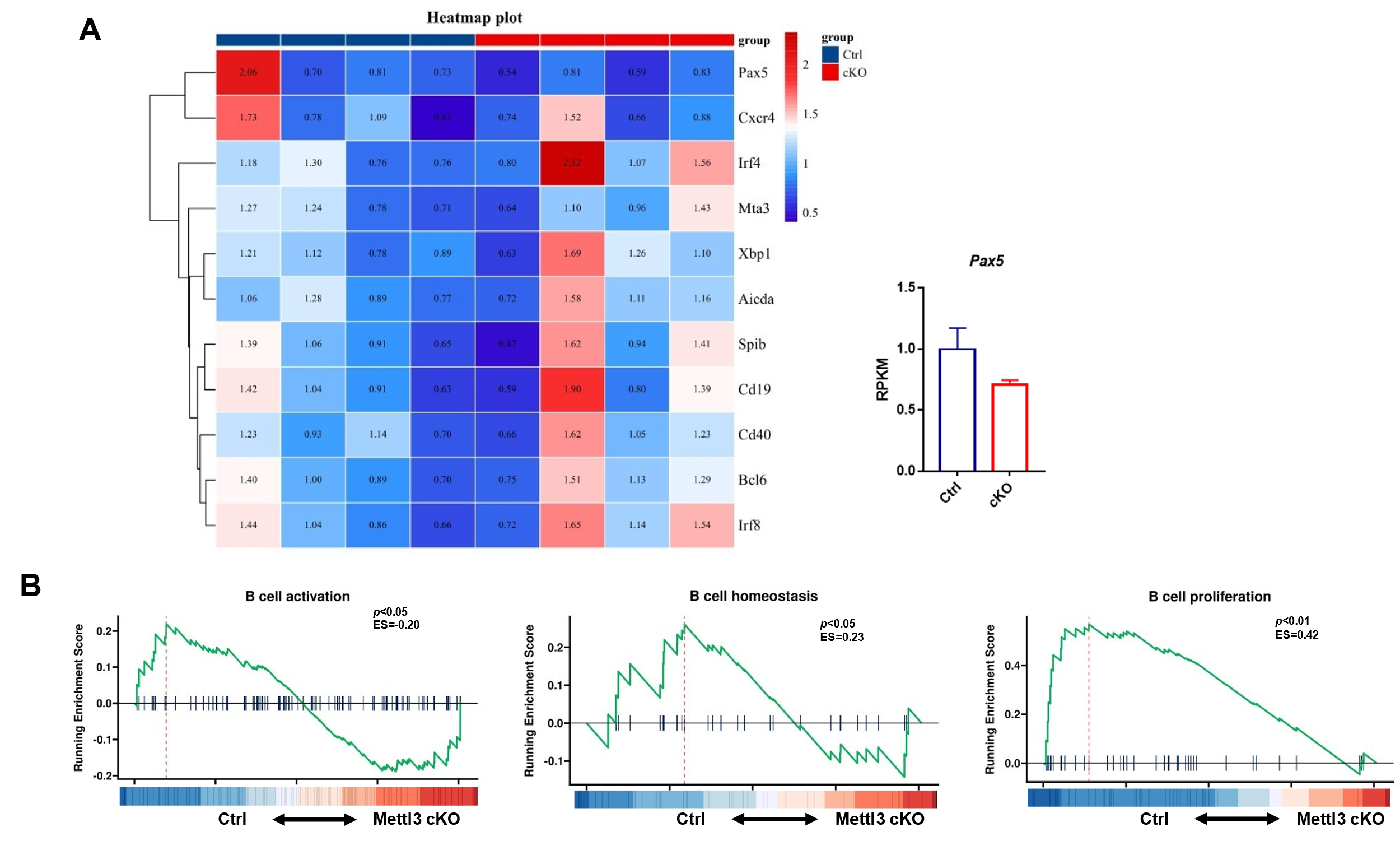


**Supplementary Figure 4. Bioinformatics analysis was used to analyze the effect of *Mettl3* knockout in B cells on B cell-related genes and pathways.**

A. Heatmap of B cell related gene expression and effect on *Pax5* after *Mettl3* knockout in GC B cells. (GSE180359)

B. Gene set enrichment analysis after *Mettl3* knockout in B cells. B cell proliferation (p<0.01, ES=0.42). B cell homeostasis (p<0.05, ES=0.23). B cell activation (p<0.05, ES=-0.20).


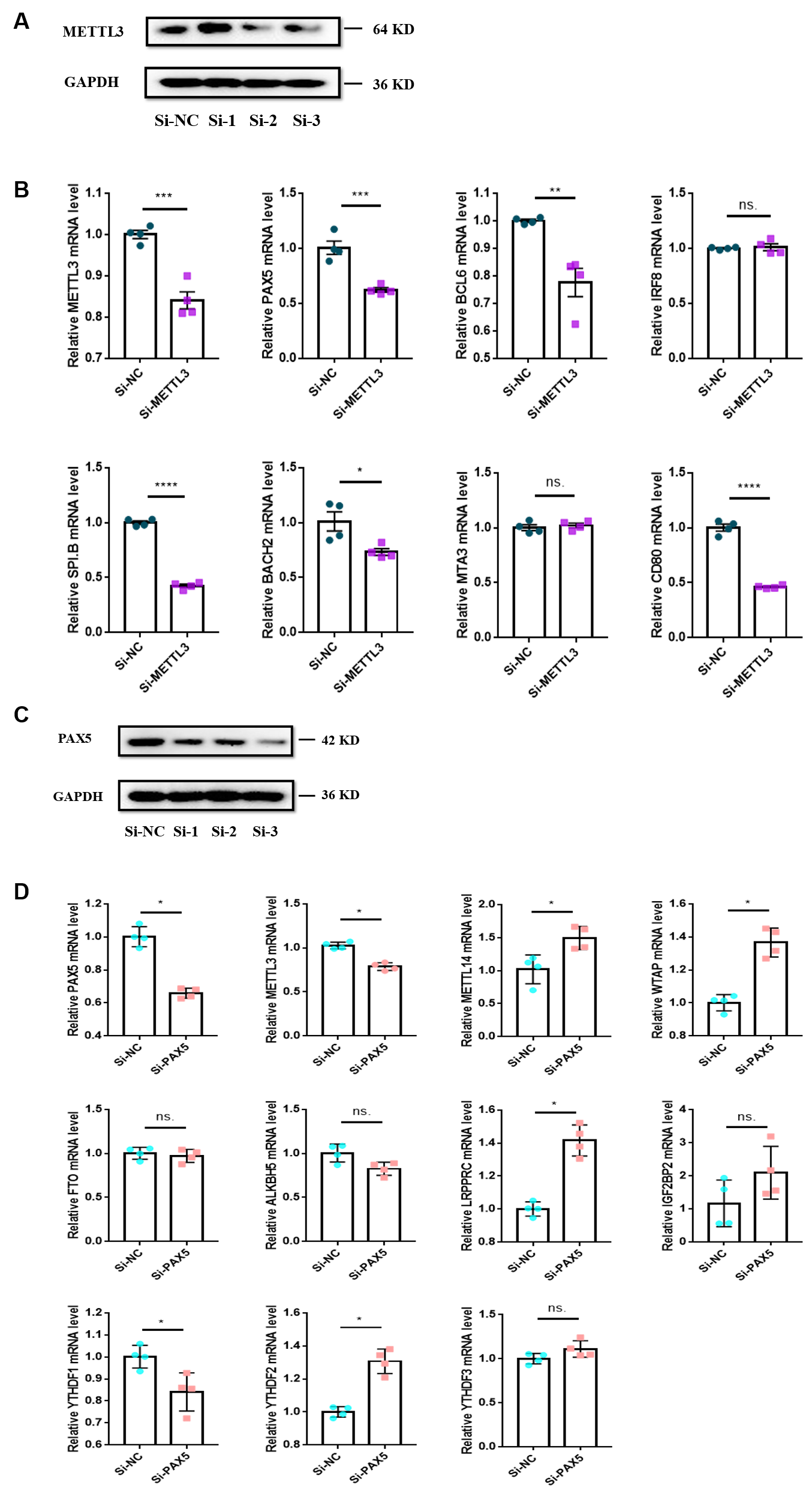


**Supplementary Figure 5. Knockdown of *METTL3* or *PAX5* in Raji B cells.**

A. Verification of the interference effect of *METTL3* siRNA fragment.

B. After knockdown of METTL3 in Raji B cells, the mRNA expression levels of *PAX5*, *BCL6*, *SPI.B*, *BACH2* and *CD80* were decreased, but the mRNA expression levels of *IRF8* and *MTA3* were not changed.

C. Verification of the interference effect of *PAX5* siRNA fragment.

D. After PAX5 knockdown in Raji cells, the mRNA expression levels of *METTL3* and *YTHDF1* decreased, while the mRNA expression levels of *METTL14*, *WTAP*, *LRPPRC* and *YTHDF2* increased. The mRNA expression levels of *FTO*, *ALKBH5*, *IGF2BP2* and *YTHDF3* did not change.

n = 4. Data are presented as mean ± SEM. **p* < 0.05, ***p* < 0.01, ****p* < 0.001, ****, *p* < 0.0001, ns.: no significance, by two-tailed unpaired Student’s t tests.
